# The Natural Pesticide Dihydrorotenone Induces Human Plasma Cell Apoptosis by Triggering Endoplasmic Reticulum Stress and Activating p38 Signaling Pathway

**DOI:** 10.1371/journal.pone.0069911

**Published:** 2013-07-26

**Authors:** Jieyu Zhang, Juan Tang, Biyin Cao, Zubin Zhang, Jie Li, Aaron D. Schimmer, Sudan He, Xinliang Mao

**Affiliations:** 1 Cyrus Tang Hematology Center, Soochow University, Suzhou, China; 2 Ontario Cancer Institute, University of Toronto, Toronto, Ontario, Canada; 3 Department of Pharmacology, College of Pharmacy, Soochow University, Suzhou, China; Duke University Medical Center, United States of America

## Abstract

Dihydrorotenone (DHR) is a natural pesticide widely used in farming industry, such as organic produces. DHR is a potent mitochondrial inhibitor and probably induces Parkinsonian syndrome, however, it is not known whether DHR is toxic to other systems. In the present study, we evaluated the cytotoxicity of DHR on human plasma cells. As predicted, DHR impaired mitochondrial function by decreasing mitochondrial membrane potential in plasma cells. Because mito-dysfunction leads to unfolded protein response (UPR) and endoplasmic reticulum (ER) stress, we examined the signature proteins in ER stress, including GRP78, ATF4, and CHOP. After DHR treatment, these proteins were significantly upregulated. It is reported that activation of the mitogen-activated protein kinases p38 and JNK are involved in endoplasmic reticulum stress. However, in the subsequent study, DHR was found to activate p38 but not the JNK signaling. When pre-treated with p38 inhibitor SB203580, activation of p38 and cell apoptosis induced by DHR was partially blocked. Thus, we found that DHR induced human plasma cell death by activating the p38 but not the JNK signaling pathway. Because plasma cells are very important in the immune system, this study provided a new insight in the safety evaluation of DHR application.

## Introduction

Organic food is becoming more and more popular in today’s green living style. Because utilization of synthetic pesticide, plant growth hormone, and chemical fertilizers are prohibited from organic produces, natural alternatives such as rotenone become the choice. Rotenone is a natural product extracted from the seeds and stems of several plants, such as the jicama vines. Rotenone could be quickly transformed into several major metabolites, one of which is dihydrorotenone (DHR). DHR is produced by the catalytic hydrogenation of the side chain of rotenone with elimination of the double bond. Like rotenone, DHR is also active as a pesticide but is much less toxic. The oral LD_50_ of rotenone is 300–500 mg/kg for rats, while it is more than 2.5 g/kg for DHR [1]. Mechanistic investigations suggested that DHR bound to and inhibited the complex I in the electron transport chain, thus inhibiting mitochondrial function and decreasing ATP production [2]. Inhibition of mitochondrial function and disruption of the energy supply lead to insect death. Although DHR is non-toxic in rats, it induces Parkinsonian syndrome in the long-term treated mice [1], which suggesting DHR is toxic to neural cells. However, whether DHR is toxic to other types of cells is not clear.

The blood system is the major target of most xenobiotics and blood cells have been developed as an efficient tool to evaluate the safety of xenobiotics. For example, gene expression profiling in monocytes exposed to xenobiotics has been proposed as a potential prediction method to evaluate toxicity of chemicals [3]. B cells and immune systems are sensitive to foreign substances, thus B cells are the major subjects for immunotoxicity study. Primary human B cell models have been established to assess the sensitivity of antibody responses to modulation by xenobiotics [4]. In the present study, we evaluated the cytotoxicity of DHR on human plasma cells, and explored the underlying molecular mechanisms. It demonstrated that DHR induced plasma cell apoptosis which was probably resulted from mitochondrial dysfunction and endoplasmic reticulum (ER) stress via activating the p38 MAP kinase. This study demonstrated that DHR is toxic to human plasma cells. Because plasma cells are a key player in the immune system and more exposure to DHR is expected with the increasing need of organic produces, caution should be taken in the assessment of DHR safety.

## Materials and Methods

### Cells and Reagents

Human plasma cell lines (KMS11, OPM2) were originally derived from patients of plasma cell neoplasm (multiple myeloma), and were generously provided by Dr. Keith Stewart, Mayo Clinic, Scottsdale, Arizona)[5]–[7]. LP1, RPMI-8226, U266 cell lines were purchased from American Type Culture Collection (ATCC). All cells were grown in Iscove’s Modified Dulbecco’s medium (IMDM, Thermo Scientific HyClone) supplemented with 10% fetal bovine serum (Invitrogen), penicillin (100 units/ml), and streptomycin (100 µg/ml) in an incubator humidified with 95% air and 5% CO_2_ at 37°C. DHR was purchased from Maybridge Chemicals, UK. SB203580 and z-VAD-fmk were purchased from Beyotime Biotechnology Institute, Nantong, China.

### Analysis of Apoptotic Cells by Flow Cytometry

Plasma cell lines LP1, KMS11, OPM2, and U266 were plated in 24-well plates (Wuxi Nest Biotechnology Co., Ltd, Wuxi, China), and treated with DHR for 24 h followed by staining with Annexin V-fluorescein isothiocyanate (Annexin V-FITC) and propidium iodide (PI, Biouniquer, Nanjing, China) according to the manufacturer’s instructions. Cells were then incubated for 10 min in dark before being subject to analysis on a flow cytometer (FACSCalibur, Becton Dickinson) as reported previously [8]. To analyze whether caspase activation is involved in DHR-induced plasma cell apoptosis, KMS11 and LP1 cells were treated in the presence of pan-caspase inhibitor, followed by evaluation on caspase-3 activation by Western blotting and plasma cell apoptosis by Annexin V/PI double staining and flow cytometry.

### Measurement of Mitochondrial Membrane Potential

LP1 cells were treated with DHR for 0, 10 or 20 µM for 24 h, or 10 µM for 0.5 to 24 h. Cells were then washed in phosphate buffered saline (PBS) and incubated with 25 nM tetramethylrhodamine methyl ester (TMRM, Promega) or co-incubated with 25 nM TMRM and 20 µg/ml Annexin V-FITC (Roche) in IMDM for 15 min at 37°C. The TMRM and Annexin V-FITC fluorescence were analyzed by flow cytometry.

### Immunoblotting

After treatment, human plasma cells were washed in cold PBS and lysed in a cell lysis buffer containing 50 mM Tris-HCI, pH 7.4, 1% NP-40, 0.5% Na-deoxycholate, and 0.1% SDS, 150 mM NaCl, 2 mM EDTA, and 50 mM NaF. For the detection of phosphoproteins, 1 mM sodium orthovanadate was added to the lysis buffer. The lysates were then sonicated followed by centrifugation at 13,000×g at 4°C for 20 min. The supernatants were collected and subject for protein concentration determination by the BCA Protein Assay Kit (Beyotime). Equal amount of proteins were fractionated by SDS–polyacrylamide gel electrophoresis. Proteins were then transferred onto a PVDF membrane (Millipore). The membranes were blocked with 5% non-fat milk or 5% bovine serum albumin (BSA) in Tris-buffered saline-Triton X-100 (TBST) for 1 h at room temperature and subsequently incubated with primary antibodies overnight at 4°C. After wash with TBST, the blots were incubated with a horseradish peroxidase conjugated secondary antibody for 1 h followed by washing with TBST. The blots were visualized by an enhanced chemoluminescence (ECL) detection system (Beyotime). Primary antibody against GRP78 was purchased from Santa Cruz Biotechnology Inc.; ATF4 antibody was purchased from BD Pharmagin; Bcl-2, Mcl-1, p-p38, p-JNK, and CHOP were purchased from Cell Signaling Technology (CST). Caspase-3, -8, -9 and -12, β-actin, and anti–mouse immunoglobulin G (IgG) and anti–rabbit IgG horseradish peroxidase conjugated antibody were purchased from R&D Systems. Bim and PARP were purchased from Abcam, China. GAPDH was purchased from Abgent, China.

### Statistical Analysis

All experiments were repeated at least 3 times. Significant analysis was performed by Student’s *t* test. Statistical significance was defined with a *p* value <0.05.

## Results

### DHR Induces Apoptosis of Human Plasma Cells

**Figure 1:**
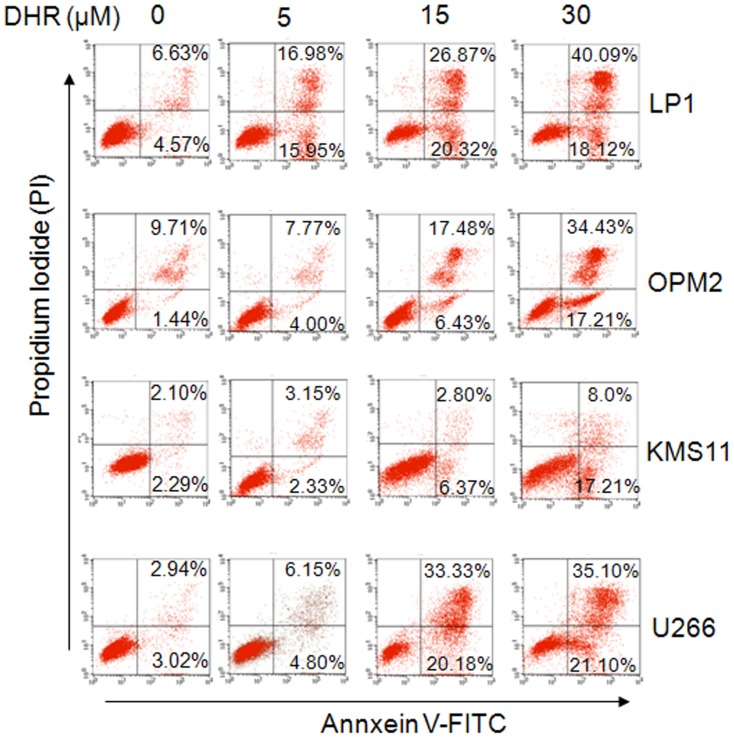
DHR induces human plasma cell apoptosis. Human plasma cell lines LP1, OPM2, KMS11 and U266 were treated with DHR for 24 h. Induction of apoptosis in human plasma cells by DHR was assessed by Annexin V-FITC and propidium iodide (PI) double staining followed by analysis on a flow cytometer.

**Figure 2:**
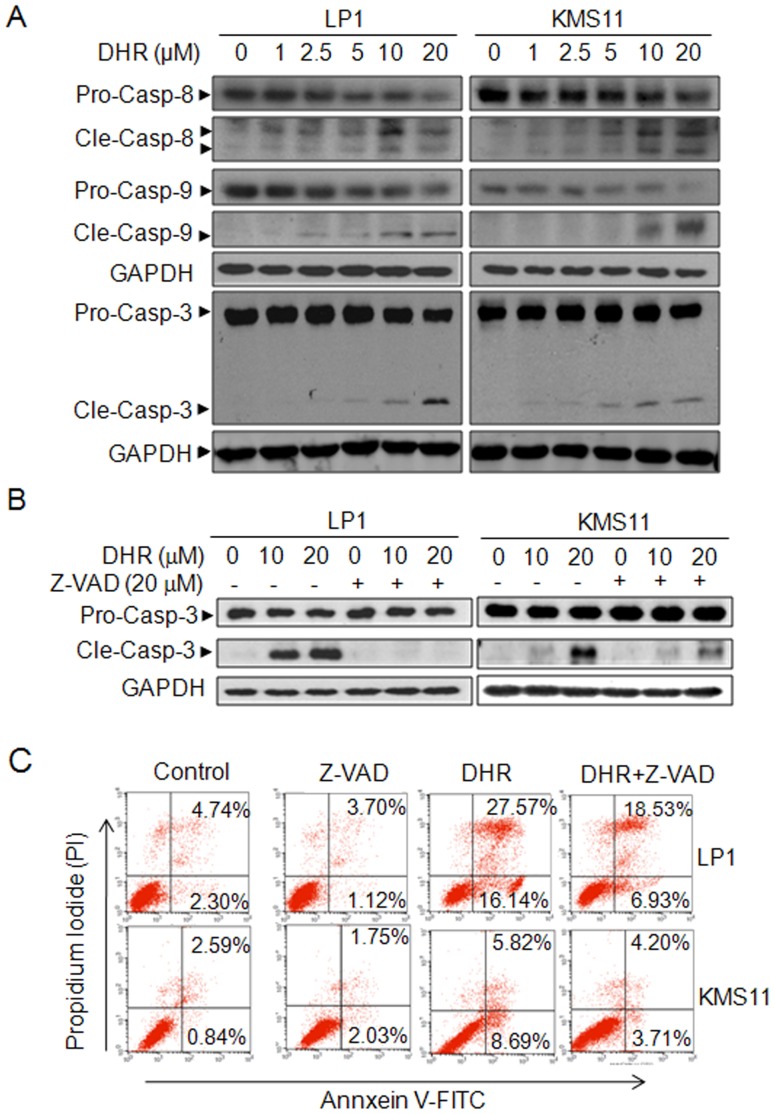
DHR induces human plasma cell death by activating apoptotic pathway. **A**, Human plasma cells LP1 and KMS11 were treated with DHR at the indicated concentrations for 24 h. Cell lysates were then prepared and subject to immunoblotting assay against apoptosis-associated proteins caspase-3, -8 and -9. GAPDH were used as a loading control. B, LP1 and KMS11 cells were treated for 24 h with DMSO, DHR, z-VAD-fmk or DHR+Z-VAD-fmk, followed by caspase-3 analysis by Western blotting. C, LP1 and KMS11 cells were treated for 24 h with DMSO, DHR, z-VAD-fmk or DHR+Z-VAD-fmk, followed by Annexin-V-FITC/PI staining and flow cytometric analyses. Pro-casp: pro-caspase; Cle-casp: cleaved caspase.

To investigate whether DHR induces human plasma cell apoptosis, human plasma cell lines LP1, OPM2, KMS11, and U266 were treated with DHR for 24 h, followed by flow cytometric analysis after being stained with Annexin V-FITC and PI. Annexin V specifically binds to the exposed phosphatidylserine on the apoptotic cell surface while PI can penetrate into dead cells and intercalates with nucleic acid. The Annexin V and PI positive fraction of all four cell lines were raised in a concentration-dependent manner (Figure 1). For example, apoptotic cells (Annexin V+) were increased from 11.2% and 11.15% in DMSO-treated control group to 47.19% and 23.91% (15 µM DHR), 58.21% and 51.64% (30 µM DHR), in LP1 and OPM2, respectively (Figure 1). Caspase-associated apoptotic pathway was then analyzed. As shown in Figure 2A, DHR activated both initiative apoptotic enzymes (caspase-8 and caspase-9) and, the executive apoptotic enzyme (caspase-3). All cleaved forms from three enzymes were increased upon DHR treatment in a concentration-dependent manner in both KMS11 and LP1 cells (Figure 2A). Because caspase activation is critical for cell apoptosis [9], to find out whether caspase activation is involved in DHR-induced apoptosis, KMS11 and LP1 cells were treated with DHR in the absence or presence of the pan-caspase inhibitor-z-VAD-fmk. Western blotting showed that DHR induced caspase-3 activation in a concentration dependent manner, but cleaved caspase-3 fragments were decreased by z-VAD-fmk (Figure 2B). Next, we evaluated cell apoptosis by Annexin-V and PI staining followed by flow cytometric analysis. It turned out that DHR increased the fraction of Annexin-V positive cells, but this fraction was significantly decreased by the addition of z-VAD-fmk (Figure 2C). For example, apoptotic cells in LP1 cell line were 43.71% induced by DHR, but it was decreased to 25.46% in the presence of z-VAD-fmk (Figure 2C). This finding was consistent with previous studies on z-VAD [10], [11]. All of these thus clearly demonstrated that caspase activation is important for DHR-induced plasma cell apoptosis.

**Figure 3:**
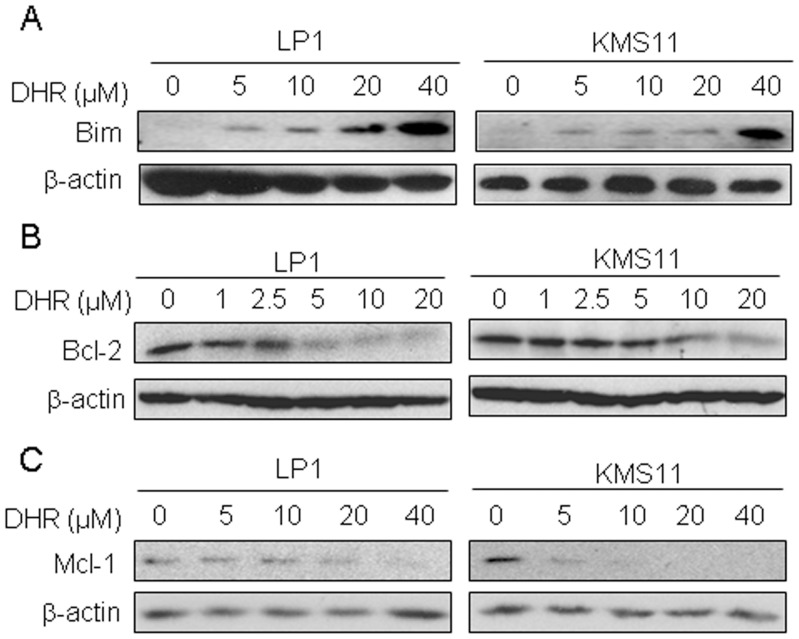
DHR dysregulates mitochondrial resident proteins in human plasma cells. Human plasma cell lines LP1 and KMS11 were treated with DHR at the indicated concentrations for 24 h. Cell lysates were then prepared and subject to immunoblotting assay against Bim (A), Bcl-2 (B) and Mcl-1 (C).β-actin was used as a loading control.

The Bcl-2 family proteins are critical regulators of apoptosis and these proteins can be divided into two main groups, anti-apoptotic (such as Bcl-2 and Mcl-1) and pro-apoptotic (such as Bim and Bax) [12]. As shown in Figures 3, pro-apoptotic Bim (Figure 3A) was induced while anti-apoptotic Bcl-2 (Figure 3B) and Mcl-1 (Figure 3C) were down-regulated in a concentration-dependent manner by DHR. Therefore, these studies suggested that DHR induced human plasma cell apoptosis.

### DHR Induces Loss of the Mitochondrial Membrane Potential in Human Plasma Cells

**Figure 4:**
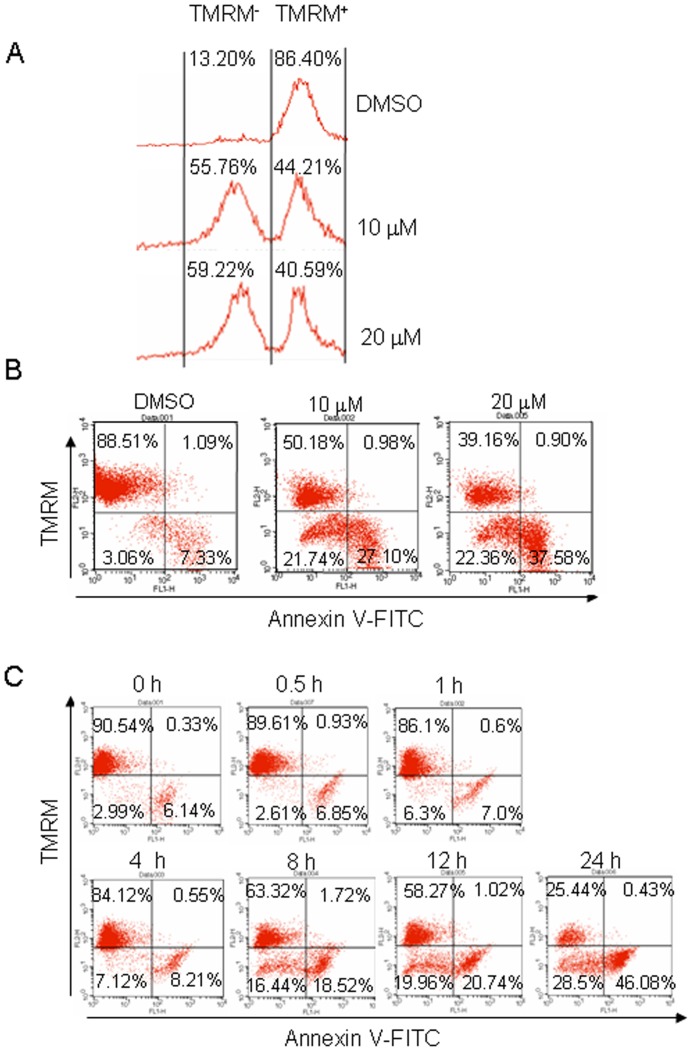
DHR leads to mitochondrial membrane potential collapse in human plasma cells. LP1 cells were treated with DMSO, 10 or 20 µM of DHR for 24 h, stained by TMRM alone (A) or in combination with Annexin V-FITC (B) followed by flow cytometric analysis. (C) LP1 cells were treated with 10 µM of DHR for indicated time periods, followed by TMRM and Annexin V-FITC staining and flow cytometric analysis.

It is reported that DHR could bind to and inhibit the Complex I (NADH:ubiquinone oxidoreductase) in the mitochondrial respiratory chain by which it blocks the electron transfer from NADH to O_2_ via the transfer of H^+^ ions (protons) across the inner mitochondrial membrane [2]. This blockade results in an insufficient proton gradient across the mitochondrial membrane, or mitochondrial membrane potential (Δψ), a sensitive marker of mitochondrial insult and its reduction has been associated with mitochondrial insult. To view this change induced by DHR, LP1 cells were treated with 10 or 20 µM of DHR for 24 h, followed by staining with tetramethylrhodamine methyl ester (TMRM), a monovalent cationic fluorescent dye widely used for measuring Δψ. Flow cytometric analysis revealed that Δψ was rapidly collapsed in response to DHR, where the potential staining cells (TMRM positive) were left-shifted (Figure 4A). Next, LP1 cells were treated for 24 h by DHR (10 or 20 µM) and co-stained with Annexin V-FITC and TMRM followed by flow cytometric analysis. As shown in 4B, addition of DHR led to increased fractions of TMRM-negative cells, especially those Annexin-V positive cells. Because TMRM is a sensitive biomarker of mitochondrion insult, to find out the time-course and relationship between mitochondrial damage and cell apoptosis, LP1 cells were incubated with 10 µM of DHR for 0.5 to 24 h followed by TMRM and Annexin V-FITC staining. Flow cytometric analysis indicated that the fractions of TMRM-negative cells were increased upon extension of incubation as shown in Figure 4C. The significant changes occurred at 8 h after DHR treatment. Interestingly, not all MMP-lost cells were Annexin V positive, suggesting mitochondrial damage was probably earlier than apoptosis.

### DHR Induces Unfolded Protein Response and Endoplasmic Reticulum Stress in Human Plasma Cells

**Figure 5:**
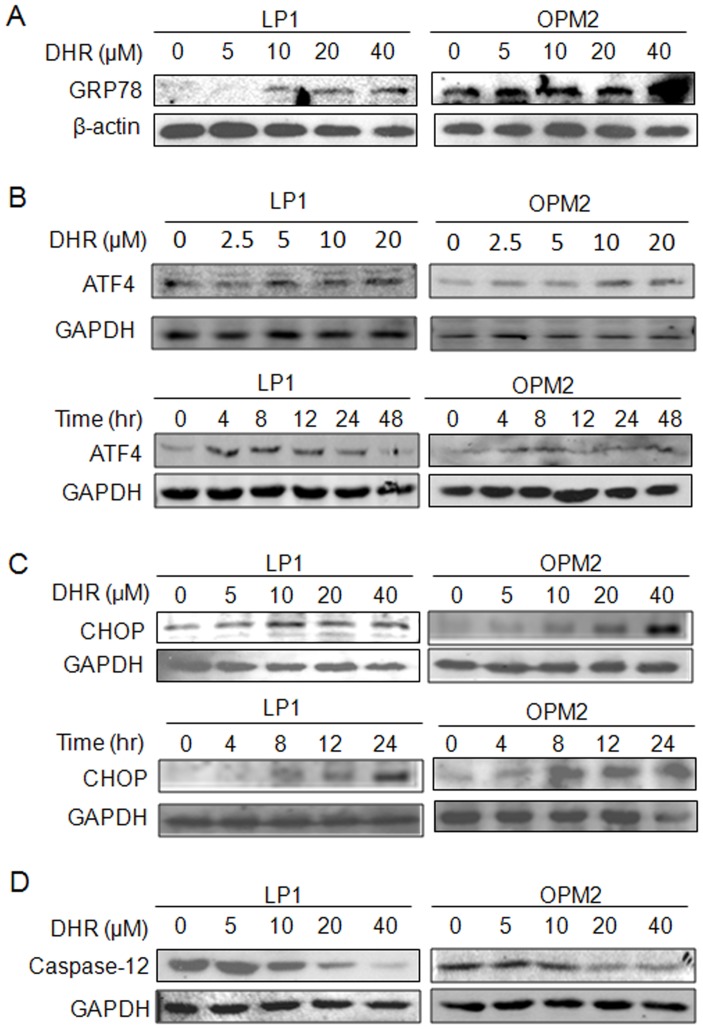
DHR activates the ER stress signaling in human plasma cells. A, LP1 and OPM2 cells were treated with DHR for 24 h, the expression of GRP78 was then detected by immunoblotting. (B) and (C) LP1 and OPM2 cells were treated DHR for different concentrations and time periods before subject to ATF4 and CHOP analysis. (D) ER-associated caspase-12 was evaluated after exposed to DHR at indicated concentrations for 24 h.

The endoplasmic reticulum (ER) contains the majority of cytochrome P450 enzymes involved in xenobiotic metabolism, as well as a number of conjugating enzymes, thus ER is involved in an array of cellular functions and plays important roles in xenobiotic metabolism and toxicity [13]. Rotenone has been found to induce ER stress in multiple cell models [14] which is proposed to be responsible for cell death [15]. As shown in the above studies, DHR was able to induce human plasma cell apoptosis which is possibly associated with mitochondrial dysfunction. But its association with ER stress is not known. To evaluate whether DHR insults endoplasmic reticulum and triggers unfolded protein response (UPR) in human plasma cells, LP1 and OPM2 were treated by DHR at increasing concentrations, followed by evaluation of the hallmarks of UPR and ER stress. We first examined glucose-regulated protein 78 (GRP78), the sensor of UPR and ER stress and a resident protein on ER membrane, which is induced when UPR is initiated. Immunoblotting analysis indicated that GRP78 was induced by DHR in a concentration-dependent manner and the highest expression level was seen at the treatment with 40 µM of DHR (Figure 5A). We next evaluated other ER stress-associated signal proteins such as ATF4 and CHOP. Western blotting analyses revealed that these two proteins were increased by DHR in both time- and concentration-dependent manners (Figures 5B and 5C). Caspase-12, the enzyme specifically responsible for the ER stress-induced apoptosis, was also decreased (Figure 5D), further suggesting that DHR induces ER stress in human plasma cells.

### DHR Activates p38 but not the c-Jun N-terminal Kinase in Human Plasma Cells

**Figure 6:**
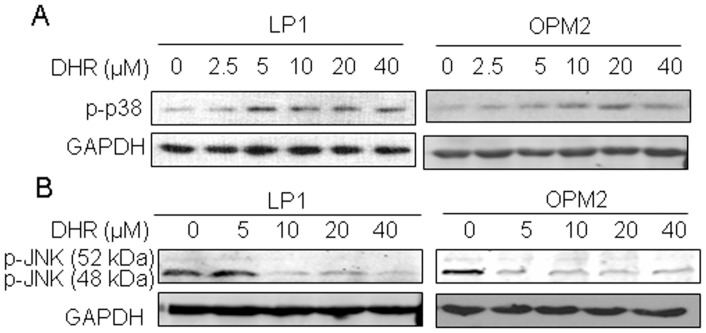
DHR activates p38 MAP kinase but suppresses JNK kinase in human plasma cells. LP1 and OPM2 cells were treated with DHR at indicated concentrations followed by immunoblotting analyses for p38 (A) and JNK (B) against specific antibodies. GAPDH was used as an internal loading control.

ER stress is highly associated with p38 and JNK signals [16], [17]. To find out the effects of DHR on these kinases associated with ER stress in human plasma cells, LP1 and OPM2 cells were treated with DHR for 24 h followed by immunoblotting using specific antibodies. As shown in Figure 6, DHR markedly induced phosphorylation of p38, but not JNK in both examined cell lines. In contrast, DHR suppressed the activation of JNK. This finding was differently from previous reports where both JNK and p38 were activated in rotenone-induced ER stress [18], suggesting that DHR probably acted in its own manner in human plasma cells.

### The p38 MAP Kinase Contributes to DHR-induced Human Plasma Cell Apoptosis

**Figure 7:**
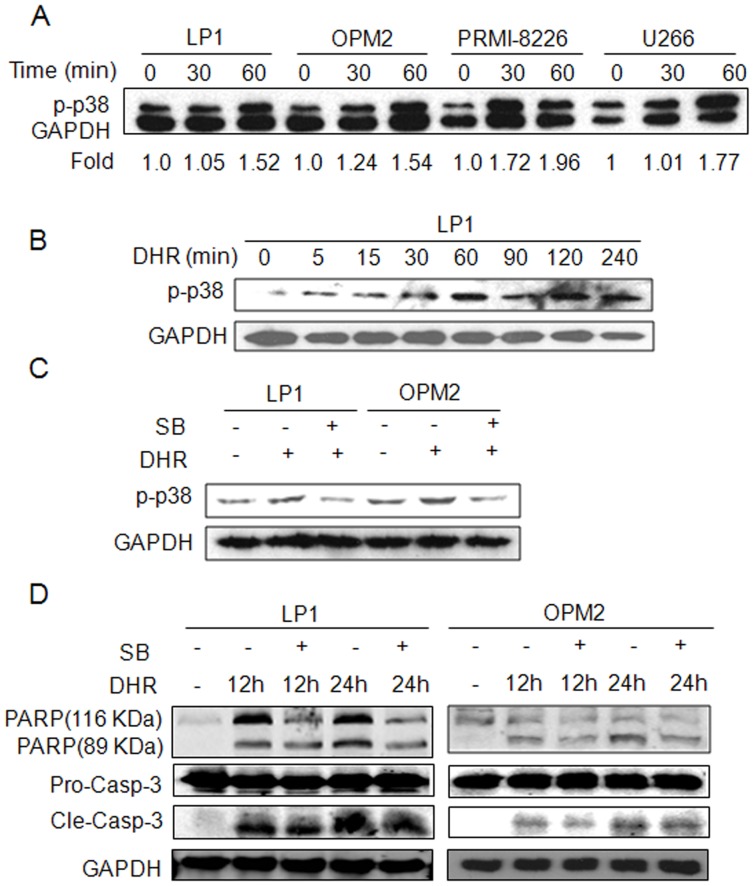
DHR activates p38 phosphorylation which is important for DHR-induced human plasma cell apoptosis. (A) Human plasma cell lines LP1, OPM2, RPMI-8226, and U266 were treated with 10 µM of DHR for 30 or 60 min, followed by p38 phosphorylation analysis. The relative phosphorylated level of p38 to GAPDH was calculated by Quality One software. (B) LP1 cells were treated by DHR at indicated time points to examine p38 activation. (C) LP1 and OPM2 were pre-treated with p38 inhibitor SB203580 (2 µM) for 30 min, followed by DHR (20 µM) for 30 min. (D) LP1 and OPM2 were pretreated with SB203580 (2 µM) for 30 min, followed by DHR (20 µM) for 12 or 24 h. Cell lysates were analyzed by specific antibodies. GAPDH was used as an internal loading control. Pro-casp-3: pro-caspase-3; Cle-casp-3: cleaved caspase-3.

To further evaluate the effect of p38 activation on DHR-induced human plasma cell apoptosis, LP1, OPM2, RPMI-8226, and U266 cells were treated with DHR for 30 or 60 min, followed by detection of p38 activation using specific antibody against phosphorylated p38. It showed that p38 was activated by DHR in all examined cell lines (Figure 7A). DHR induced p38 phosphorylation was time-dependent. As shown in Figure 7B, activated p38 signal was detected at 5 min after DHR treatment, and the highest level was seen at 240 min. Next, we evaluated the effects of DHR on cell death when p38 activation was blocked by SB203580, a specific inhibitor of p38. Pre-treatment with SB203580 attenuated activation of p38 by DHR (Figure 7C). To further evaluate the effect of p38 activation on plasma cell apoptosis, LP1 and OPM2 cells were pre-treated with SB203580 for 30 min, followed by DHR for 12 or 24 h. Immunoblotting assay demonstrated that both cleavage of PARP and caspase-3 were partly attenuated by SB203580 (Figure 7D). Because PARP and caspase-3 are key apoptotic markers [9], these results confirmed that p38 MAP kinase pathway contributed to DHR-induced plasma cell apoptosis.

## Discussion

In the above studies, we investigated the effects on DHR on human plasma cell apoptosis and underlying mechanisms. DHR is the catalytic reduction product of rotenone at the isopropylene side chain by *C. blakesleean*a [19]. Although several compounds can be isolated from rotenone metabolites, DHR is the only active one in killing pests and insects by inhibiting the electron transport chain [20]. DHR is less toxic than rotenone in the acute toxicity evaluation in rats by oral administration [1], but it also induces Parkinson’s syndrome, which suggested that DHR might be toxic in chronic exposure [1]. Because mitochondrial insult is a major factor in induction of cell death, there is no wonder that DHR can induce human plasma cell apoptosis as demonstrated in Figure 1. This apoptosis induced by DHR in human plasma cells is associated with downregulation of pro-survival proteins (such as Bcl-2 and Mcl-1) and upregulation of pro-apoptotic proteins such as Bim. Because these proteins are critical for maintenance of mitochondrial membrane integrity and membrane potential, thus DHR-induced MMP collapse further demonstrated that DHR was toxic to mitochondria.

In addition to mitochondria, ER is also an important organelle in regulating cell apoptosis. Mitochondria and ER interact both physically and functionally via the mitochondria-associated ER membrane, a linkage between the duo [21]. Mitochondria dysfunction is believed to induce UPR and ER stress featured with induced expression of GRP78 and CHOP [22]. This was also observed in DHR-treated human plasma cells. After treatment with DHR, ER stress hallmarks including GRP78, ATF4, and CHOP were induced in a concentration-dependent manner. GRP78, an ER resident molecular chaperone, binds to newly-synthesized proteins as they are translocated into the ER, and maintains them in a state competent for subsequent folding and oligomerization. Therefore, GRP78 is a central regulator of ER homeostasis and a sensor of ER stress. When unfolded or misfolded proteins are overloaded, the interaction between GRP78 and their partners is destabilized thus inducing UPR. In this study, GRP78 was markedly induced in both LP1 and OPM2 cells exposed to DHR, suggesting ER stress was initiated by DHR. Under ER stress, all PERK, ATF6 and Ire1 signals converge to induced transcription of CHOP [23], a central transcription factor in ER stress. Increased concentrations or extended treatment periods lead to more expression of CHOP by DHR (Figure 5B). We also found that DHR activated caspase-12, an initiating apoptotic enzyme responsible for ER stress-induced cell apoptosis [24]. This finding further demonstrated that ER stress was involved in DHR-induced cell death.

The mitogen-activated protein kinases (MAPKs) mainly including ERK, p38 and JNK, are involved in the development and progression of cancer and are also associated with ER stress. ERK1/2 are activated by growth factors, while p38 and JNK are mainly activated by cellular stresses [25]. The present results showed that DHR-induced activation of p38 (Figure 7), and the inhibition of p38 by SB203580 led to an ablation of DHR-induced apoptosis (Figure 7D). These results revealed a strong correlation between the level of p38 activation and sensitivity to DHR toxicity. However, DHR failed to activate the JNK signaling pathway in human plasma cells which is critical for rotenone [18], suggesting that DHR induces ER stress in a manner different from rotenone. However, activation of p38 and inactivation of JNK are possibly equally important for DHR-induced human plasma cell death because these two pathways also have antagonistic effects [26]. Recently, p38-dependent Fas-mediated apoptosis was found to be potentiated by the inhibition of JNK1/2 [27]. Down-regulation of JNK activity associated with ER stress was observed in dexamethasome-treated OCI-MY5 cells in which dexamethasome-induced apoptosis does not involve activation of JNK [28]. Therefore, the lack of activation of JNK by DHR in human plasma cells suggests that antagonistic effect of p38 and JNK in DHR-induced apoptosis.

In summary, this study demonstrated that DHR exposure leads to human plasma cell death in association with mitochondrial dysfunction, ER stress and p38 signaling. Because plasma cells are an important component of the immune system, the present study suggested that DHR probably interferes with the immune system. With the emerging need of the organic produces, exposure to DHR is also increasing, therefore, safety assessment of DHR and other rotenoids should be cautious.

## References

[1] Ambrose AM, Christensen HE, Rather LJ (1953) Toxicological and pharmacological studies on dihydrorotenone. J Am Pharm Assoc Am Pharm Assoc (Baltim) 42: 364–366. 10.1002/jps.3030420615 13069324

[2] Talpade DJ, Greene JG, Higgins DS Jr, Greenamyre JT (2000) In vivo labeling of mitochondrial complex I (NADH:ubiquinone oxidoreductase) in rat brain using [(3)H]dihydrorotenone. J Neurochem 75: 2611–2621. 11080215 10.1046/j.1471-4159.2000.0752611.x

[3] Arkusz J, Stepnik M, Sobala W, Dastych J (2010) Prediction of the contact sensitizing potential of chemicals using analysis of gene expression changes in human THP-1 monocytes. Toxicol Lett 199: 51–59. 20713136 10.1016/j.toxlet.2010.08.005

[4] Lu H, Crawford RB, North CM, Kaplan BL, Kaminski NE (2009) Establishment of an immunoglobulin m antibody-forming cell response model for characterizing immunotoxicity in primary human B cells. Toxicol Sci 112: 363–373. 19767444 10.1093/toxsci/kfp224 PMC2777081

[5] Namba M, Ohtsuki T, Mori M, Togawa A, Wada H, et al (1989) Establishment of five human myeloma cell lines. In Vitro Cell Dev Biol 25: 723–729. 2768132 10.1007/BF02623725

[6] Tiedemann RE, Mao X, Shi CX, Zhu YX, Palmer SE, et al (2008) Identification of kinetin riboside as a repressor of CCND1 and CCND2 with preclinical antimyeloma activity. J Clin Invest 118: 1750–1764. 18431519 10.1172/JCI34149 PMC2323188

[7] Katagiri S, Yonezawa T, Kuyama J, Kanayama Y, Nishida K, et al (1985) Two distinct human myeloma cell lines originating from one patient with myeloma. Int J Cancer 36: 241–246. 3926660 10.1002/ijc.2910360217

[8] Mao X, Liang SB, Hurren R, Gronda M, Chow S, et al (2008) Cyproheptadine displays preclinical activity in myeloma and leukemia. Blood 112: 760–769. 18502826 10.1182/blood-2008-02-142687

[9] Miller DK (1997) The role of the Caspase family of cysteine proteases in apoptosis. Semin Immunol 9: 35–49. 9106306 10.1006/smim.1996.0058

[10] Gao J, Liu X, Rigas B (2005) Nitric oxide-donating aspirin induces apoptosis in human colon cancer cells through induction of oxidative stress. Proc Natl Acad Sci U S A 102: 17207–17212. 16282376 10.1073/pnas.0506893102 PMC1287992

[11] Slee EA, Zhu H, Chow SC, MacFarlane M, Nicholson DW, et al (1996) Benzyloxycarbonyl-Val-Ala-Asp (OMe) fluoromethylketone (Z-VAD.FMK) inhibits apoptosis by blocking the processing of CPP32. Biochem J 315 (Pt 1): 21–24. 10.1042/bj3150021 PMC1217173 8670109

[12] Sheridan C, Martin SJ (2010) Mitochondrial fission/fusion dynamics and apoptosis. Mitochondrion 10: 640–648. 20727425 10.1016/j.mito.2010.08.005

[13] Cribb AE, Peyrou M, Muruganandan S, Schneider L (2005) The endoplasmic reticulum in xenobiotic toxicity. Drug Metab Rev 37: 405–442. 16257829 10.1080/03602530500205135

[14] Chen YY, Chen G, Fan Z, Luo J, Ke ZJ (2008) GSK3beta and endoplasmic reticulum stress mediate rotenone-induced death of SK-N-MC neuroblastoma cells. Biochem Pharmacol 76: 128–138. 18508033 10.1016/j.bcp.2008.04.010

[15] Goncalves AP, Maximo V, Lima J, Singh KK, Soares P, et al (2011) Involvement of p53 in cell death following cell cycle arrest and mitotic catastrophe induced by rotenone. Biochim Biophys Acta 1813: 492–499. 21223980 10.1016/j.bbamcr.2011.01.006 PMC3051352

[16] Chen CL, Lin CF, Chang WT, Huang WC, Teng CF, et al (2008) Ceramide induces p38 MAPK and JNK activation through a mechanism involving a thioredoxin-interacting protein-mediated pathway. Blood 111: 4365–4374. 18270325 10.1182/blood-2007-08-106336

[17] Park GB, Kim YS, Lee HK, Song H, Cho DH, et al (2010) Endoplasmic reticulum stress-mediated apoptosis of EBV-transformed B cells by cross-linking of CD70 is dependent upon generation of reactive oxygen species and activation of p38 MAPK and JNK pathway. J Immunol 185: 7274–7284. 21078900 10.4049/jimmunol.1001547

[18] Deng YT, Huang HC, Lin JK (2010) Rotenone induces apoptosis in MCF-7 human breast cancer cell-mediated ROS through JNK and p38 signaling. Mol Carcinog 49: 141–151. 19777565 10.1002/mc.20583

[19] Sariaslani FS, Rosazza JP (1983) Microbial Transformations of Natural Antitumor Agents: Products of Rotenone and Dihydrorotenone Transformation by Cunninghamella blakesleeana. Appl Environ Microbiol 45: 616–621. 16346210 10.1128/aem.45.2.616-621.1983 PMC242333

[20] Higgins DS Jr, Greenamyre JT (1996) [3H]dihydrorotenone binding to NADH: ubiquinone reductase (complex I) of the electron transport chain: an autoradiographic study. J Neurosci 16: 3807–3816. 8656275 10.1523/JNEUROSCI.16-12-03807.1996 PMC6578603

[21] Simmen T, Lynes EM, Gesson K, Thomas G (2010) Oxidative protein folding in the endoplasmic reticulum: tight links to the mitochondria-associated membrane (MAM). Biochim Biophys Acta 1798: 1465–1473. 20430008 10.1016/j.bbamem.2010.04.009 PMC2885528

[22] Lee JW, Kim WH, Yeo J, Jung MH (2010) ER stress is implicated in mitochondrial dysfunction-induced apoptosis of pancreatic beta cells. Mol Cells 30: 545–549. 21340672 10.1007/s10059-010-0161-5

[23] Szegezdi E, Logue SE, Gorman AM, Samali A (2006) Mediators of endoplasmic reticulum stress-induced apoptosis. EMBO Rep 7: 880–885. 16953201 10.1038/sj.embor.7400779 PMC1559676

[24] Szegezdi E, Fitzgerald U, Samali A (2003) Caspase-12 and ER-stress-mediated apoptosis: the story so far. Ann N Y Acad Sci 1010: 186–194. 15033718 10.1196/annals.1299.032

[25] Keshet Y, Seger R (2010) The MAP kinase signaling cascades: a system of hundreds of components regulates a diverse array of physiological functions. Methods Mol Biol 661: 3–38. 20811974 10.1007/978-1-60761-795-2_1

[26] Ichijo H (1999) From receptors to stress-activated MAP kinases. Oncogene 18: 6087–6093. 10557099 10.1038/sj.onc.1203129

[27] Tourian L Jr, Zhao H, Srikant CB (2004) p38alpha, but not p38beta, inhibits the phosphorylation and presence of c-FLIPS in DISC to potentiate Fas-mediated caspase-8 activation and type I apoptotic signaling. J Cell Sci 117: 6459–6471. 15572410 10.1242/jcs.01573

[28] Chauhan D, Pandey P, Ogata A, Teoh G, Treon S, et al (1997) Dexamethasone induces apoptosis of multiple myeloma cells in a JNK/SAP kinase independent mechanism. Oncogene 15: 837–843. 9266970 10.1038/sj.onc.1201253

