# Correction: The Natural Pesticide Dihydrorotenone Induces Human Plasma Cell Apoptosis by Triggering Endoplasmic Reticulum Stress and Activating p38 Signaling Pathway

**DOI:** 10.1371/journal.pone.0234162

**Published:** 2020-06-02

**Authors:** Jieyu Zhang, Juan Tang, Biyin Cao, Zubin Zhang, Jie Li, Aaron D. Schimmer, Sudan He, Xinliang Mao

After publication of this article [1], similarities were noted between the following figure panels:

- Fig 1, FACS plots shown for OPM2/5μM DHR and KMS11/5μM DHR
- β-actin blots for KMS11 in Fig 3A and for OPM2 in Fig 5A

**Fig 1:**
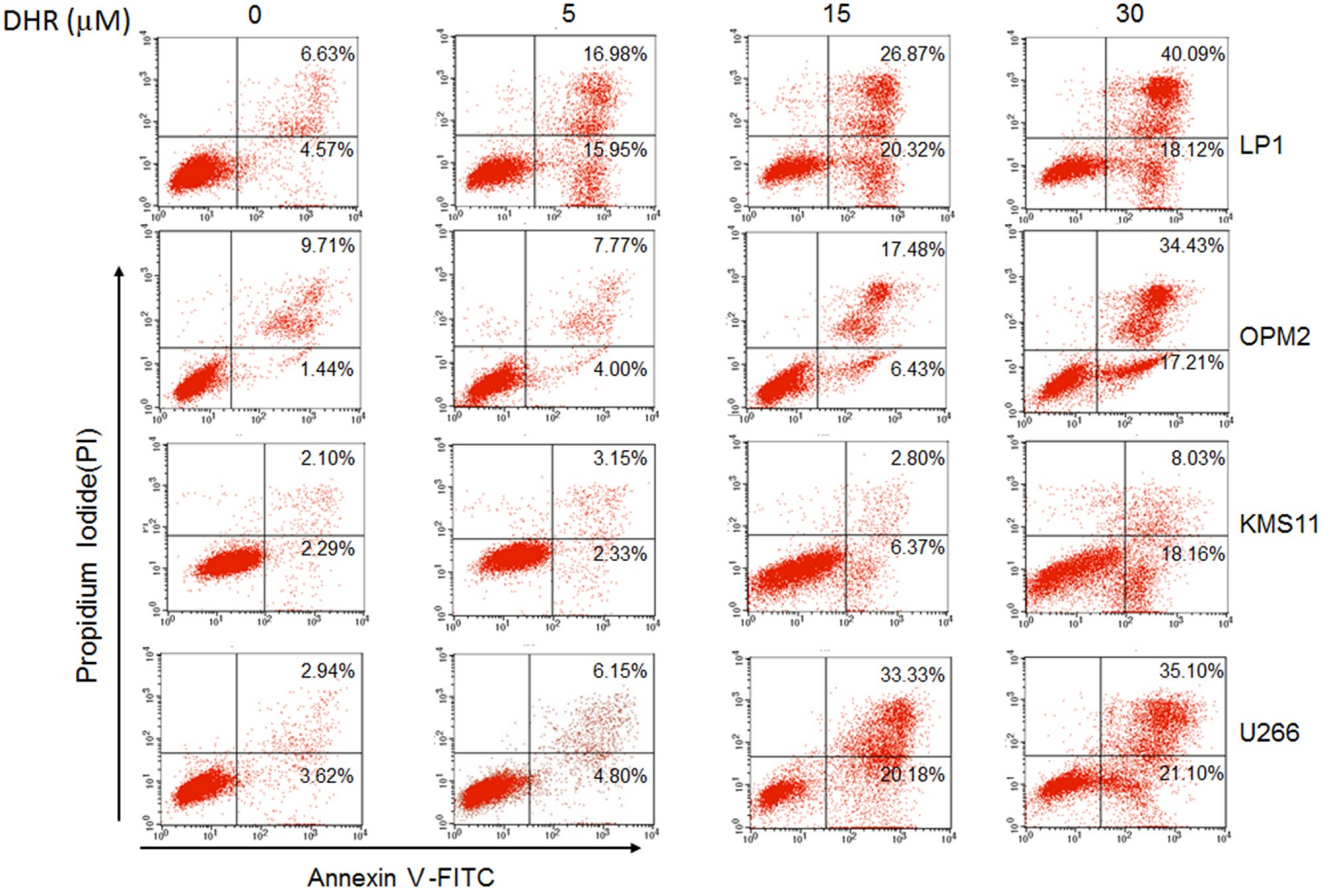
DHR induces human plasma cell apoptosis. Human plasma cell lines LP1, OPM2, KMS11 and U266 were treated with DHR for 24 h. Induction of apoptosis in human plasma cells by DHR was assessed by Annexin V-FITC and propidium iodide (PI) double staining followed by analysis on a flow cytometer.

An error was made in preparing Fig 1 such that the OPM2 FACS plot is duplicated in the KMS11 panel. In addition, there are errors in the percentages reported on the U266/0 μM DHR and KMS11/30 μM DHR panels. A corrected Fig 1 is provided with this Correction; dot plots and quantitative data for these FACS analyses are in S1 File.

The experiments in Fig 3A and 3C are mislabeled: these experiments were conducted in OPM2 cells, not KMS11 cells, and used the same blots as the GPR78 experiment shown in Fig 5A. Hence, the same β-actin control blot applied for Fig 3A and 5A. The β-actin data shown in the indicated panels were obtained by cutting the blot used for the Bim experiment shown in Fig 3A and then probing the resultant membrane strips (from the same blot) for β-actin, GAPDH, and Bim. The GPR78 experiment was done subsequently by stripping the Bim blot. The available original data for the Fig 3A and 3C experiments are in S1–S3 Files. Both β-actin and GAPDH controls were included in these experiments but only a single loading control was included in each Fig. The control data shown in Fig 3C are mislabeled, these panels show GAPDH results. The LP1/β-actin blot in the original published Fig 3A does not match the original data provided for this experiment and this is addressed in the updated Fig 3.

In addition, incorrect percentages are reported in Fig 2C for the right quadrants of the KMS11/DHR+Z-VAD plot; in Fig 4A for DMSO, TMRM^+^; and in Fig 4C for the lower left quadrant of the 24 h panel. These errors have been corrected in the updated Figs with this notice. In the new version of Fig 4, the histograms in Fig 4A have been replaced to align with available raw data from the original experiments. Traces, dot plots, and quantitative data supporting these FACS results are in S1 File. The raw data files (.fcs) to support the flow cytometry results are no longer available.

**Fig 2:**
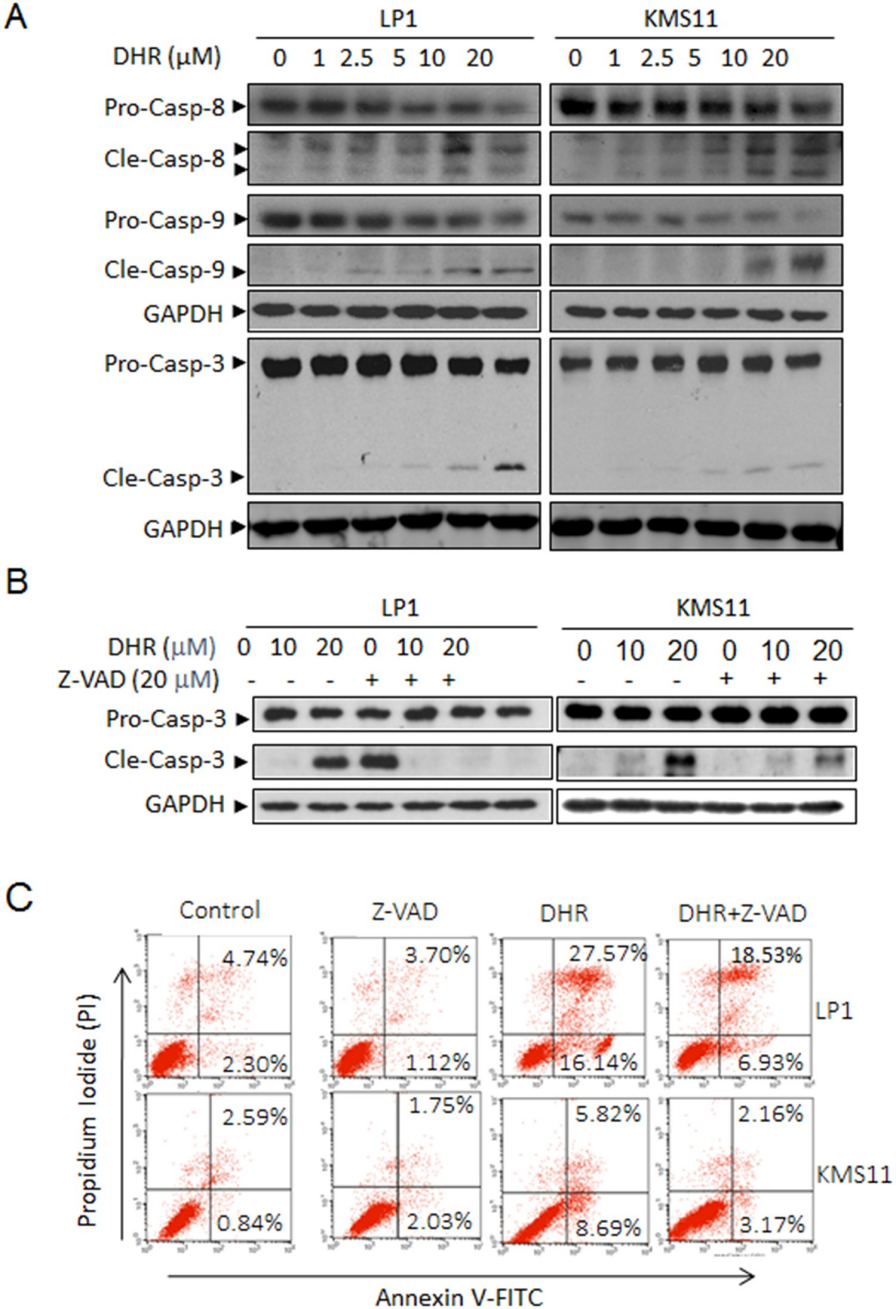
DHR induces human plasma cell death by activating apoptotic pathway. A, Human plasma cells LP1 and KMS11 were treated with DHR at the indicated concentrations for 24 h. Cell lysates were then prepared and subject to immunoblotting assay against apoptosis-associated proteins caspase-3, -8 and -9. GAPDH was used as a loading control. B, LP1 and KMS11 cells were treated for 24 h with DMSO, DHR, z-VAD-fmk or DHR+Z-VAD-fmk, followed by caspase-3 analysis by Western blotting. C, LP1 and KMS11 cells were treated for 24 h with DMSO, DHR, z-VAD-fmk or DHR+Z-VAD-fmk, followed by Annexin-V-FITC/PI staining and flow cytometric analyses. Pro-casp: pro-caspase; Cle-casp: cleaved caspase.

**Fig 3:**
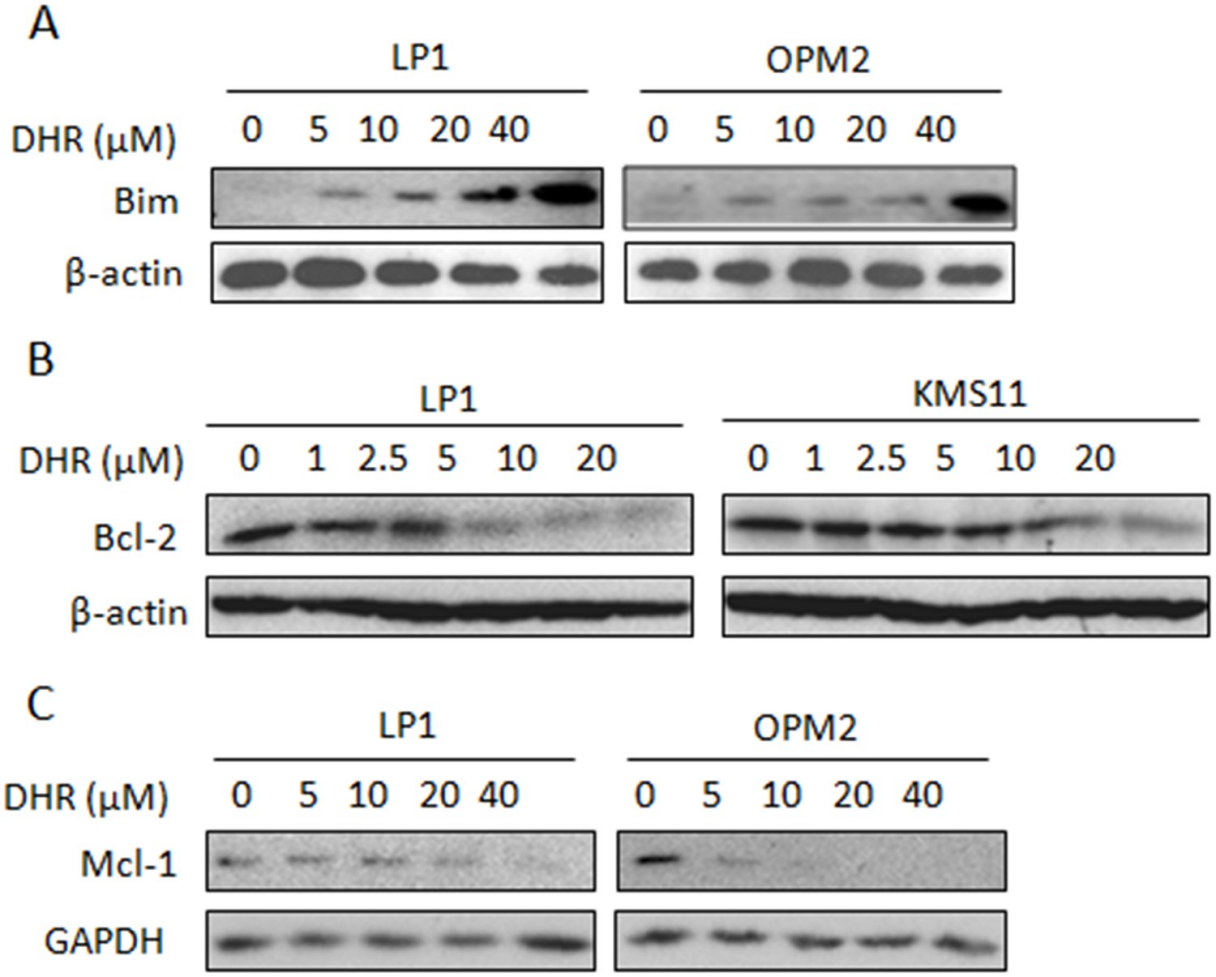
DHR dysregulates mitochondrial resident proteins in human plasma cells. Human plasma cell lines were treated with DHR at the indicated concentrations for 24 h. Cell lysates were then prepared and subject to immunoblotting assay against Bim (A), Bcl-2 (B) and Mcl-1 (C).

**Fig 4:**
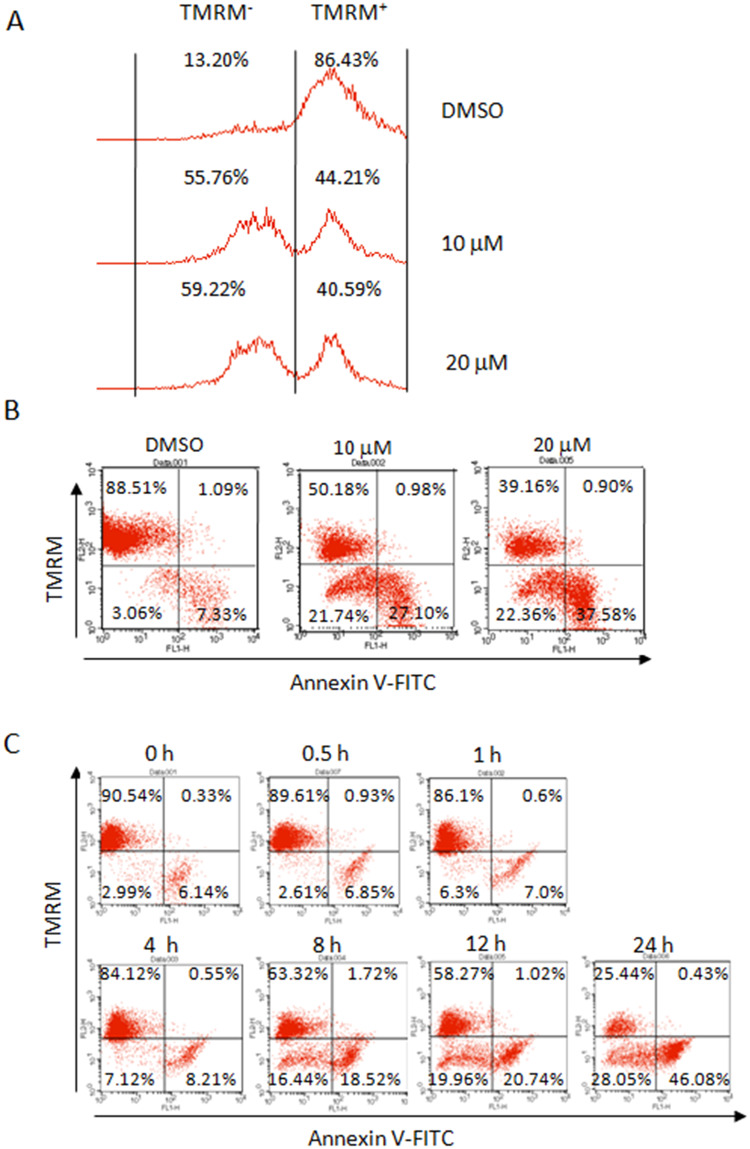
DHR leads to mitochondrial membrane potential collapse in human plasma cells. LP1 cells were treated with DMSO, 10 or 20 μM of DHR for 24 h, stained by TMRM alone (A) or in combination with Annexin V-FITC (B) followed by flow cytometric analysis. (C) LP1 cells were treated with 10 μM of DHR for indicated time periods, followed by TMRM and Annexin V-FITC staining and flow cytometric analysis.

All available data to support other results reported in the article are in S1 File. As noted in the article’s Materials and Methods, all experiments were repeated (biological replicates, using fresh samples) at least three times.

A member of *PLOS ONE*’s Editorial Board reviewed the corrected figures and advised that whilst such mistakes should not have occurred, the corrections address the errors.

The authors apologize for the errors in the published article.

## Supporting information

S1 File Underlying data to support Figs 1, 2, 3A, 3C and 5A. As indicated in the file, due to mistakes in curation, raw blots are no longer available for the following: cleaved caspase-8 and 9 in Fig 2A; GAPDH for Caspase-3 in Fig 2A; Caspase-3 in KMS11 for Fig 2B; GAPDH for LP1 time course experiment in Fig 5C; CHOP and GAPDH for OPM2 concentration-dependent assays in Fig 5C. Note, the Fig 3C blots for OPM2 are erroneously labelled in S1 File as representing KMS11 experiments.
(PDF)

S2 File Raw blots to support Fig 3A. (PDF)

S3 File Raw blots to support Fig 3C. (PDF)

## References

[1] Zhang J, Tang J, Cao B, Zhang Z, Li J, Schimmer AD, et al (2013) The Natural Pesticide Dihydrorotenone Induces Human Plasma Cell Apoptosis by Triggering Endoplasmic Reticulum Stress and Activating p38 Signaling Pathway. PLoS ONE 8(7): e69911 10.1371/journal.pone.0069911 23922854 PMC3724796

