# Supplementary material for: Correction: The Natural Pesticide Dihydrorotenone Induces Human Plasma Cell Apoptosis by Triggering Endoplasmic Reticulum Stress and Activating p38 Signaling Pathway
Source: PLoS One. 2020 Jun 2;15(6):e0234162. doi: 10.1371/journal.pone.0234162 (PMC7266331; doi:10.1371/journal.pone.0234162)
Supplement: S1 File — As indicated in the file, due to mistakes in curation, raw blots are no longer available for the following: cleaved caspase-8 and 9 in Fig 2A; GAPDH for Caspase-3 in Fig 2A; Caspase-3 in KMS11 for Fig 2B; GAPDH for LP1 time course experiment in Fig 5C; CHOP and GAPDH for OPM2 concentration-dependent assays in Fig 5C. Note, the Fig 3C blots for OPM2 are erroneously labelled in S1 File as representing KMS11 experiments. (PDF) [file pone.0234162.s001.pdf]

The Raw data regarding the paper “The natural pesticide dihydrorotenone induces human plasma cell apoptosis by triggering endoplasmic reticulum stress and activating p38 signaling pathway”, ***PLoS One***. 2013; 8(7):e69911. doi: 10.1371/journal.pone.0069911.

Flow analysis on LP1 ---Original data

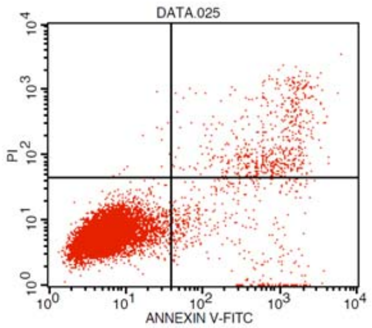

Quadrant Statistics

Sample ID: lp1 btb 0
Gate: G1
Gated Events: 9485
Total Events: 10000
X Parameter: ANNEXIN V-FITC (Log)
Y Parameter: PI (Log)

Quad	Events	% Gated	% Total
UL	12	0.13	0.12
UR	629	6.63	6.29
LL	8411	88.68	84.11
LR	433	4.57	4.33

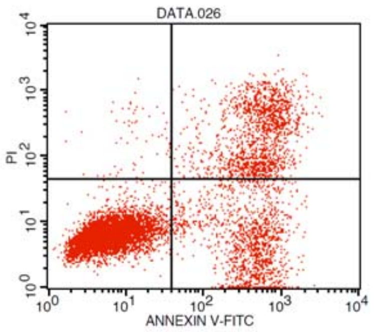

Quadrant Statistics

Sample ID: lp1 btb 5
Gate: G1
Gated Events: 9026
Total Events: 10000
X Parameter: ANNEXIN V-FITC (Log)
Y Parameter: PI (Log)

Quad	Events	% Gated	% Total
UL	59	0.65	0.59
UR	1533	16.98	15.33
LL	5994	66.41	59.94
LR	1440	15.95	14.40

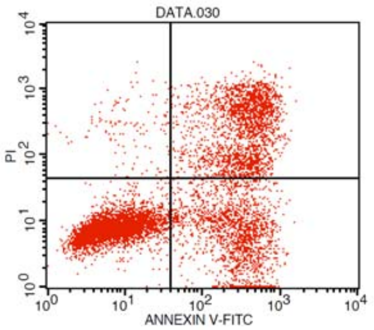

Quadrant Statistics

Sample ID: lp1 btb 15
Gate: G1
Gated Events: 8415
Total Events: 10000
X Parameter: ANNEXIN V-FITC (Log)
Y Parameter: PI (Log)

Quad	Events	% Gated	% Total
UL	124	1.47	1.24
UR	2261	26.87	22.61
LL	4320	51.34	43.20
LR	1710	20.32	17.10

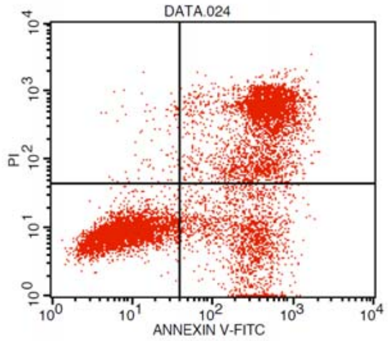

Quadrant Statistics

Sample ID: lp1 btb 30
Gate: G1
Gated Events: 8924
Total Events: 10000
X Parameter: ANNEXIN V-FITC (Log)
Y Parameter: PI (Log)

Quad	Events	% Gated	% Total
UL	78	0.87	0.78
UR	3578	40.09	35.78
LL	3651	40.91	36.51
LR	1617	18.12	16.17

Figure 1, FLOW original readout

Flow analysis on OPM2---Original data

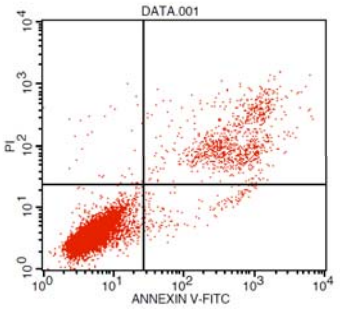

Quadrant Statistics

File: DATA.001
Sample ID: OPM2-0
Acquisition Date: 17-Jul-11
Gate: G1
Gated Events: 9684
Total Events: 10000
X Parameter: ANNEXIN V-FITC (Log)
Y Parameter: PI (Log)
Quad Location: 26, 23

Quad	Events	% Gated	% Total
UL	31	0.32	0.31
UR	940	9.71	9.40
LL	8574	88.54	85.74
LR	139	1.44	1.39

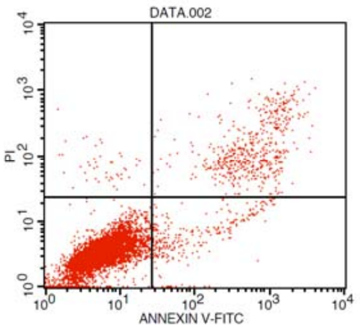

Quadrant Statistics

File: DATA.002
Sample ID: OPM2-5
Acquisition Date: 17-Jul-11
Gate: G1
Gated Events: 6899
Total Events: 10000
X Parameter: ANNEXIN V-FITC (Log)
Y Parameter: PI (Log)
Quad Location: 26, 23

Quad	Events	% Gated	% Total
UL	45	0.65	0.45
UR	536	7.77	5.36
LL	6042	87.58	60.42
LR	276	4.00	2.76

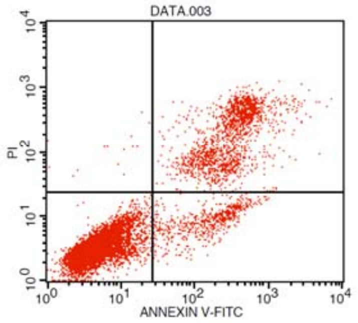

Quadrant Statistics

File: DATA.003
Sample ID: OPM2-15
Acquisition Date: 17-Jul-11
Gate: G1
Gated Events: 9521
Total Events: 10000
X Parameter: ANNEXIN V-FITC (Log)
Y Parameter: PI (Log)
Quad Location: 26, 23

Quad	Events	% Gated	% Total
UL	17	0.18	0.17
UR	1664	17.48	16.64
LL	7228	75.92	72.28
LR	612	6.43	6.12

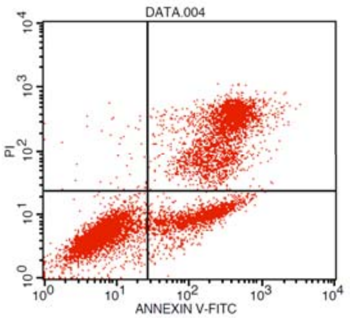

Quadrant Statistics

File: DATA.004
Sample ID: OPM2-30
Acquisition Date: 17-Jul-11
Gate: G1
Gated Events: 8990
Total Events: 10000
X Parameter: ANNEXIN V-FITC (Log)
Y Parameter: PI (Log)
Quad Location: 26, 23

Quad	Events	% Gated	% Total
UL	42	0.47	0.42
UR	3095	34.43	30.95
LL	4306	47.90	43.06
LR	1547	17.21	15.47

Figure 1, FLOW original readout

Flow analysis on KMS11---Original data

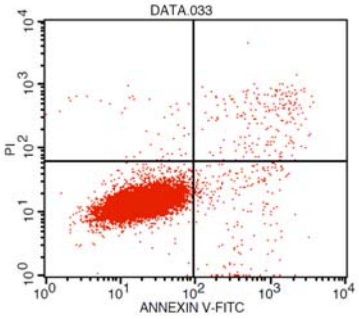

Quadrant Statistics

File: DATA.033
Sample ID: KMS11 0
Panel: Untitled Acquisition Tube List
Acquisition Date: 01-Jun-11
Gate: G1
Gated Events: 9703
Total Events: 10000
X Parameter: ANNEXIN V-FITC (Log)
Y Parameter: PI (Log)

Quad	Events	% Gated	% Total
UL	46	0.47	0.46
UR	204	2.10	2.04
LL	9231	95.14	92.31
LR	222	2.29	2.22

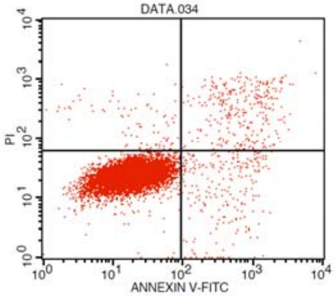

Quadrant Statistics

File: DATA.034
Sample ID: KMS11 5
Panel: Untitled Acquisition Tube List
Acquisition Date: 01-Jun-11
Gate: G1
Gated Events: 9651
Total Events: 10000
X Parameter: ANNEXIN V-FITC (Log)
Y Parameter: PI (Log)

Quad	Events	% Gated	% Total
UL	83	0.86	0.83
UR	304	3.15	3.04
LL	9039	93.66	90.39
LR	225	2.33	2.25

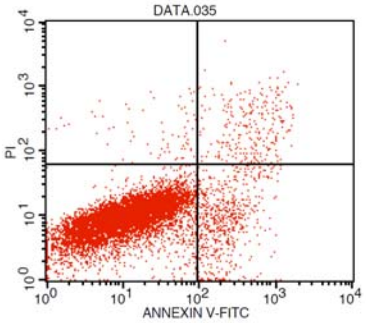

Quadrant Statistics

File: DATA.035
Sample ID: KMS11 15
Panel: Untitled Acquisition Tube List
Acquisition Date: 01-Jun-11
Gate: G1
Gated Events: 9253
Total Events: 10000
X Parameter: ANNEXIN V-FITC (Log)
Y Parameter: PI (Log)

Quad	Events	% Gated	% Total
UL	68	0.73	0.68
UR	259	2.80	2.59
LL	8337	90.10	83.37
LR	589	6.37	5.89

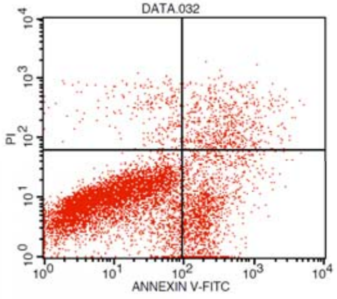

Quadrant Statistics

File: DATA.032
Sample ID: KMS11 30
Panel: Untitled Acquisition Tube List
Acquisition Date: 01-Jun-11
Gate: G1
Gated Events: 7908
Total Events: 10000
X Parameter: ANNEXIN V-FITC (Log)
Y Parameter: PI (Log)

Quad	Events	% Gated	% Total
UL	236	2.98	2.36
UR	635	8.03	6.35
LL	5601	70.83	56.01
LR	1436	18.16	14.36

Figure 1, FLOW original readout

Flow analysis on U266---Original data

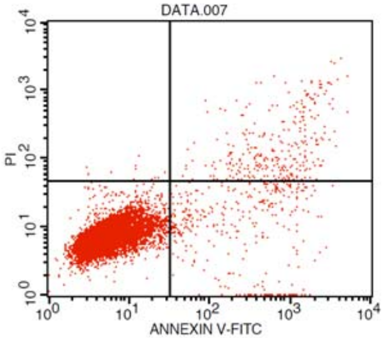

Quadrant Statistics

File: DATA.007
Sample ID: U266-0
Tube: Untitled
Acquisition Date: 11-Jun-11

Quad	Events	% Gated	% Total
UL	12	0.12	0.12
UR	289	2.94	2.89
LL	9166	93.31	91.66
LR	356	3.62	3.56

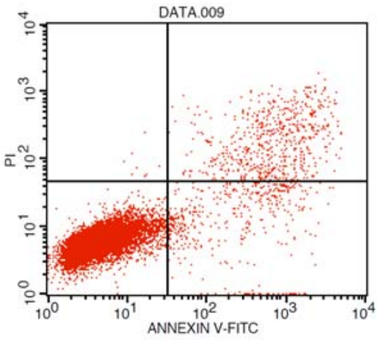

Quadrant Statistics

File: DATA.009
Sample ID: U266-5
Tube: Untitled
Acquisition Date: 11-Jun-11

Quad	Events	% Gated	% Total
UL	10	0.10	0.10
UR	605	6.15	6.05
LL	8752	88.95	87.52
LR	472	4.80	4.72

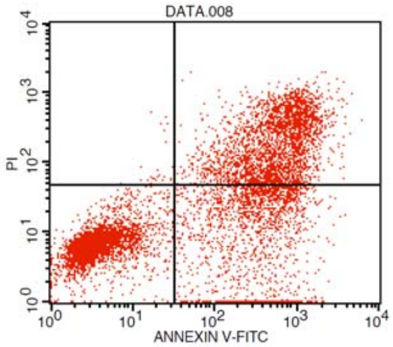

Quadrant Statistics

File: DATA.008
Sample ID: U266-15
Tube: Untitled
Acquisition Date: 11-Jun-11

Quad	Events	% Gated	% Total
UL	24	0.28	0.24
UR	2866	33.33	28.66
LL	3973	46.21	39.73
LR	1735	20.18	17.35

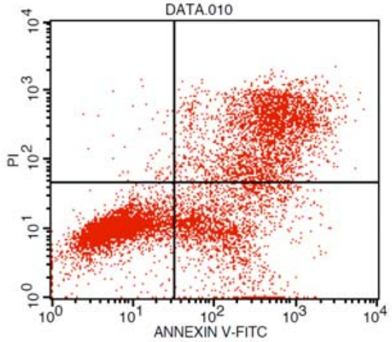

Quadrant Statistics

File: DATA.010
Sample ID: U266-30
Tube: Untitled
Acquisition Date: 11-Jun-11

Quad	Events	% Gated	% Total
UL	57	0.62	0.57
UR	3243	35.10	32.43
LL	3990	43.18	39.90
LR	1950	21.10	19.50

Figure 1, FLOW original readout

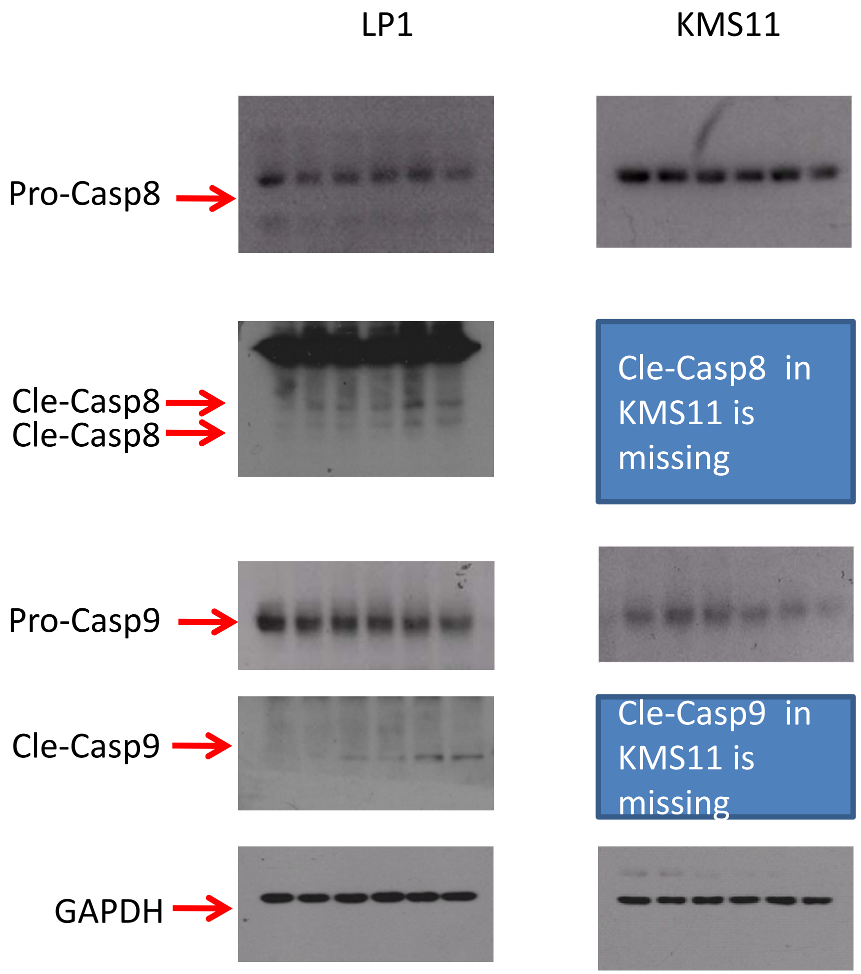
LP1
KMS11
Pro-Casp8
Cle-Casp8
Cle-Casp8
Cle-Casp8 in KMS11 is missing
Pro-Casp9
Cle-Casp9
Cle-Casp9 in KMS11 is missing
GAPDH

Fig. 2A

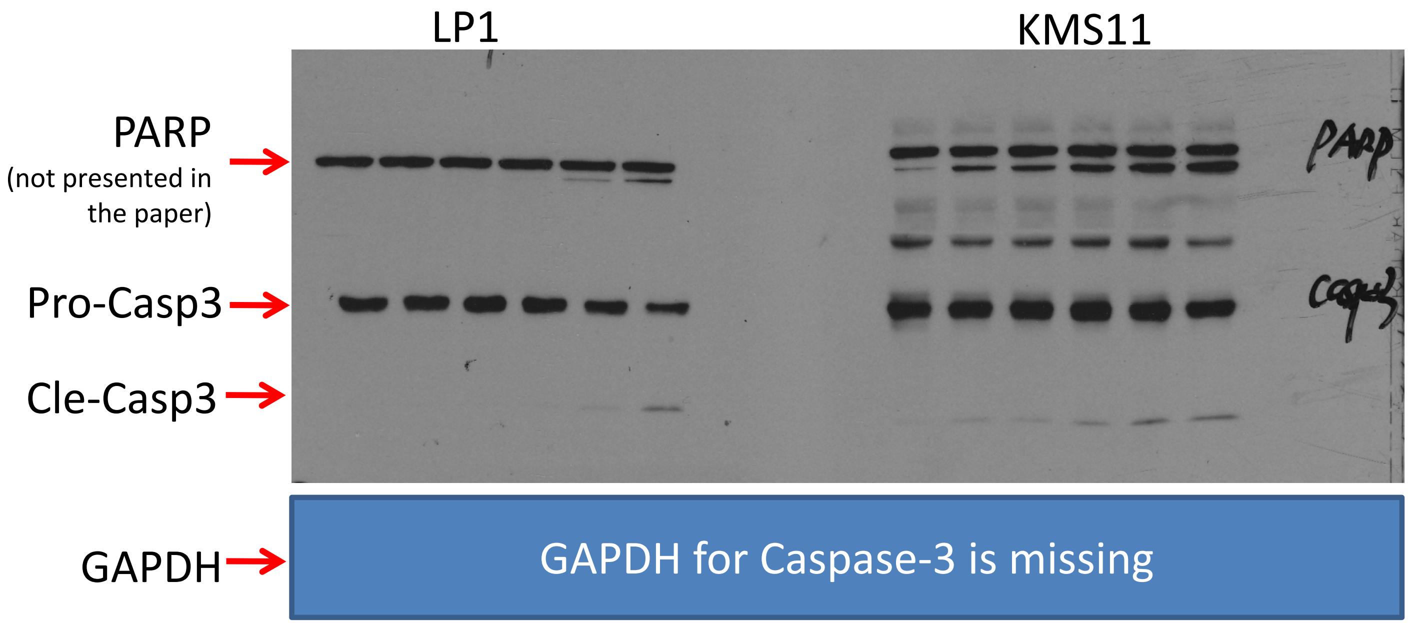
LP1
KMS11
PARP
(not presented in the paper)
Pro-Casp3
Cle-Casp3
GAPDH
GAPDH for Caspase-3 is missing

Fig. 2A

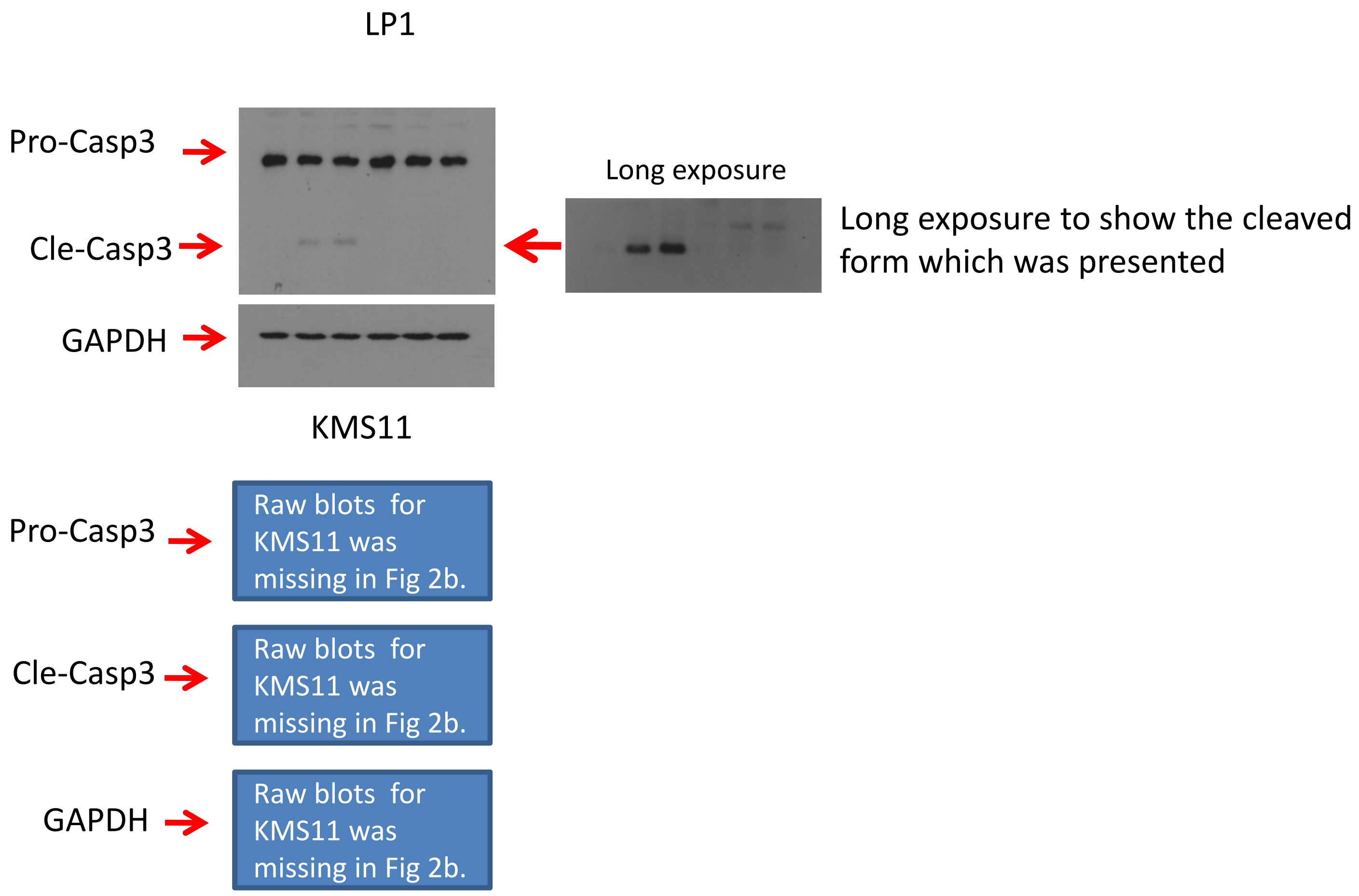
LP1
Pro-Casp3
Cle-Casp3
GAPDH
Long exposure
Long exposure to show the cleaved form which was presented
KMS11
Pro-Casp3
Raw blots for KMS11 was missing in Fig 2b.
Cle-Casp3
Raw blots for KMS11 was missing in Fig 2b.
GAPDH
Raw blots for KMS11 was missing in Fig 2b.

Fig. 2B

Figure 2C for LP1

DHR only

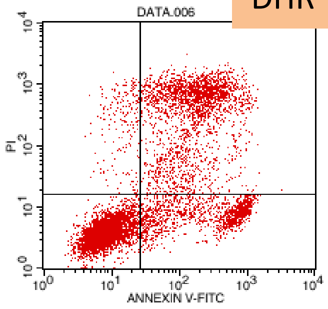

Sample ID: LP1-dhr

Quad	Events	% Gated	% Total
UL	225	2.60	2.25
UR	2382	27.57	23.82
LL	4639	53.69	46.39
LR	1395	16.14	13.95

Z-VAD only

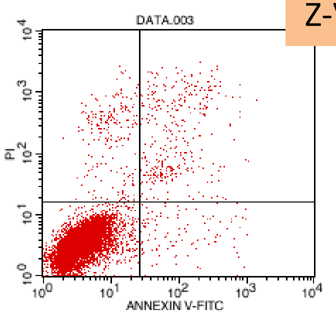

Sample ID: LP1-z-vad

Quad	Events	% Gated	% Total
UL	217	2.24	2.17
UR	358	3.70	3.58
LL	8990	92.94	89.90
LR	108	1.12	1.08

Z-VAD + DHR

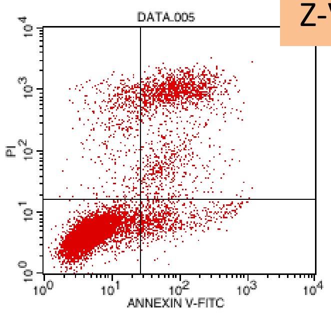

Sample ID: LP1-dhr+z-vad

Quad	Events	% Gated	% Total
UL	457	5.01	4.57
UR	1690	18.53	16.90
LL	6340	69.53	63.40
LR	632	6.93	6.32

Control only

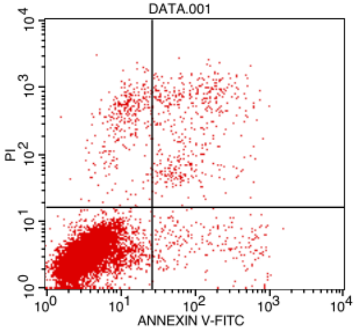

Sample ID: LP1-ctl

Quad	Events	% Gated	% Total
UL	299	3.14	2.99
UR	451	4.74	4.51
LL	8555	89.83	85.55
LR	219	2.30	2.19

Figure 2C for KMS11

DHR only

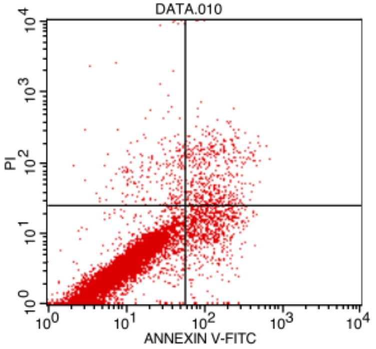

Sample ID: kms dhr

Quad	Events	% Gated	% Total
UL	213	2.58	2.13
UR	481	5.82	4.81
LL	6857	82.91	68.57
LR	719	8.69	7.19

Control only

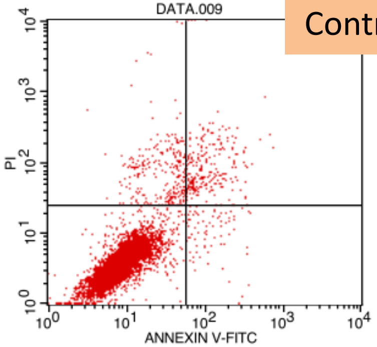

Sample ID: kms ctl

Quad	Events	% Gated	% Total
UL	261	2.75	2.61
UR	246	2.59	2.46
LL	8921	93.83	89.21
LR	80	0.84	0.80

Z-VAD only

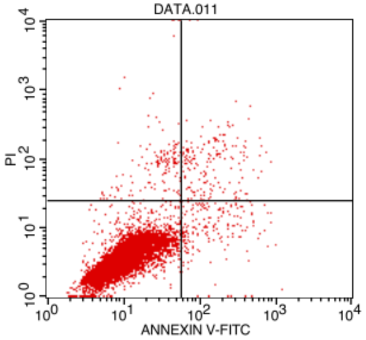

Sample ID: kms z-vad

Quad	Events	% Gated	% Total
UL	135	1.40	1.35
UR	169	1.75	1.69
LL	9160	94.82	91.60
LR	196	2.03	1.96

Z-VAD + DHR

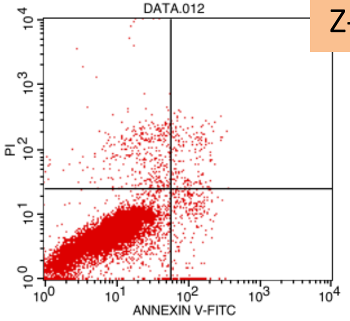

Sample ID: kms z-vad+dhr

Quad	Events	% Gated	% Total
UL	345	4.20	3.45
UR	177	2.16	1.77
LL	7425	90.47	74.25
LR	260	3.17	2.60

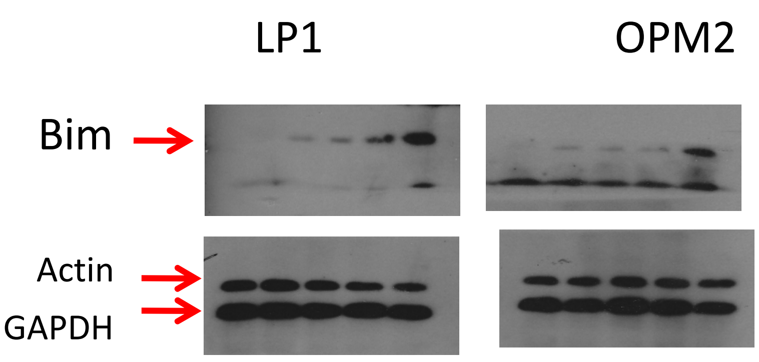

Fig. 3A

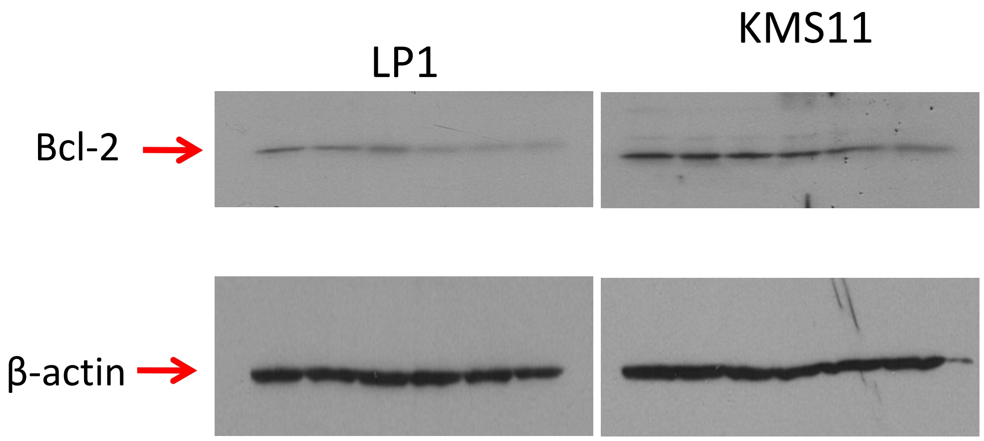

Fig. 3B

Figure 4A. DHR leads to mitochondrial membrane potential collapse stained by TRAM only

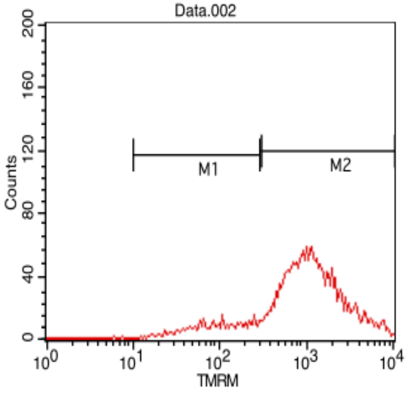

Histogram Statistics

File: Data.002
Sample ID: LP1-SS-0
Tube: Untitled
Panel: Untitled Acquisition Tube List
Acquisition Date: 18-Oct-12
Gate: G1
Gated Events: 9700
Total Events: 10000

Marker	Left, Right	Events	% Gated	% Total
All	1, 9910	9700	100.00	97.00
M1	10, 286	1280	13.20	12.80
M2	300, 9910	8384	86.43	83.84

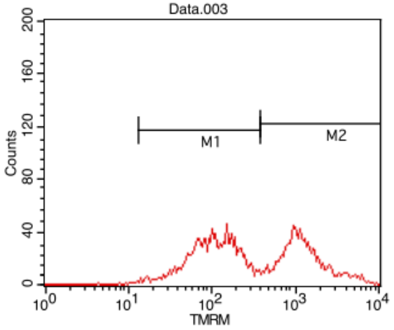

Histogram Statistics

File: Data.003
Log Data Units: Linear Values
Sample ID: LP1-SS-10DHR
Acquisition Date: 18-Oct-12
Gate: G1
Gated Events: 9150
Total Events: 10000
X Parameter: TMRM (Log)

Marker	Left, Right	Events	% Gated	% Total
All	1, 9910	9150	100.00	91.50
M1	13, 375	5102	55.76	51.02
M2	375, 9910	4045	44.21	40.45

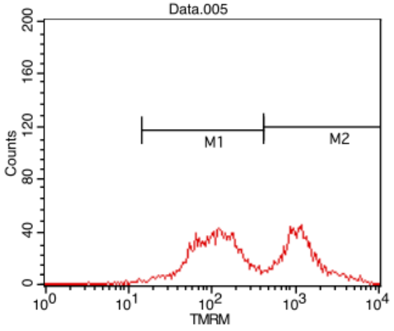

Histogram Statistics

File: Data.005
Log Data Units: Linear Values
Sample ID: LP1-SS-20DHR
Acquisition Date: 18-Oct-12
Gate: G1
Gated Events: 9270
Total Events: 10000
X Parameter: TMRM (Log)

Marker	Left, Right	Events	% Gated	% Total
All	1, 9910	9270	100.00	92.70
M1	14, 410	5490	59.22	54.90
M2	410, 9910	3763	40.59	37.63

Figure 4B. DHR leads to mitochondrial membrane potential collapse stained by TRAM (FL2) and Annexin V (FL1)

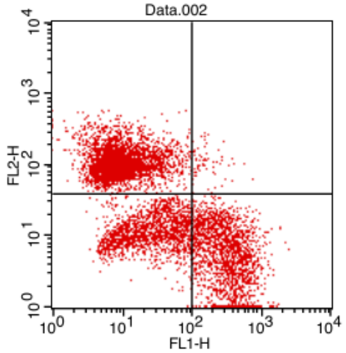

Quadrant Statistics

File: Data.002
Log Data Units: Linear Values
Sample ID: LP1-DS-10DHR
Gate: G1
Gated Events: 7033
Total Events: 10000
X Parameter: FL1-H (Log)
Y Parameter: FL2-H (Log)

Quad	Events	% Gated	% Total
UL	3529	50.18	35.29
UR	69	0.98	0.69
LL	1529	21.74	15.29
LR	1906	27.10	19.06

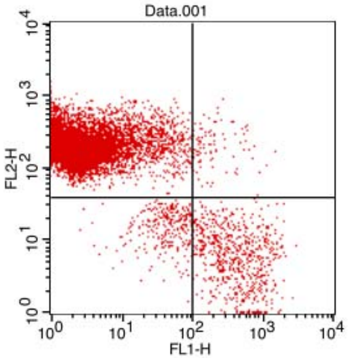

Quadrant Statistics

File: Data.001
Log Data Units: Linear Values
Sample ID: LP1-DS-0
Gate: G1
Gated Events: 8881
Total Events: 10000
X Parameter: FL1-H (Log)
Y Parameter: FL2-H (Log)

Quad	Events	% Gated	% Total
UL	7861	88.51	78.61
UR	97	1.09	0.97
LL	272	3.06	2.72
LR	651	7.33	6.51

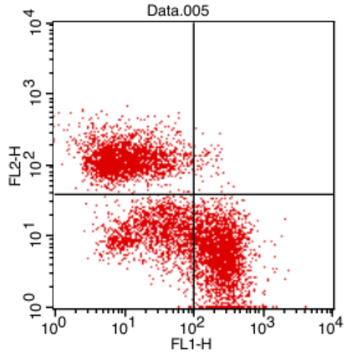

Quadrant Statistics

File: Data.005
Log Data Units: Linear Values
Sample ID: LP1-DS-5SB+20DHR
Gate: G1
Gated Events: 7030
Total Events: 10000
X Parameter: FL1-H (Log)
Y Parameter: FL2-H (Log)

Quad	Events	% Gated	% Total
UL	2753	39.16	27.53
UR	63	0.90	0.63
LL	1572	22.36	15.72
LR	2642	37.58	26.42

Figure 4C.

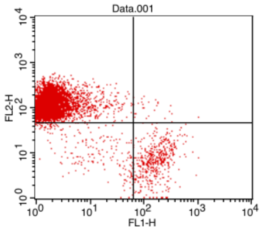

Quadrant Statistics

File: Data.001
Sample ID: LP1-DS-0
Gate: G1
Gated Events: 7835
Total Events: 10000
X Parameter: FL1-H (Log)
Y Parameter: FL2-H (Log)

Quad	Events	% Gated	% Total
UL	7094	90.54	70.94
UR	26	0.33	0.26
LL	234	2.99	2.34
LR	481	6.14	4.81

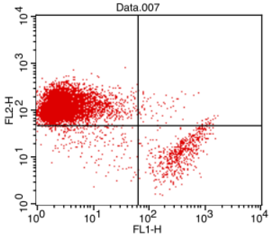

Quadrant Statistics

File: Data.007
Sample ID: LP1-DS-0.54h-20uMDHR
Gate: G1
Gated Events: 7959
Total Events: 10000
X Parameter: FL1-H (Log)
Y Parameter: FL2-H (Log)

Quad	Events	% Gated	% Total
UL	7132	89.61	71.32
UR	74	0.93	0.74
LL	208	2.61	2.08
LR	545	6.85	5.45

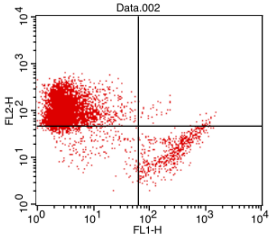

Quadrant Statistics

File: Data.002
Sample ID: LP1-DS-1h-20uMDHR
Gate: G1
Gated Events: 7798
Total Events: 10000
X Parameter: FL1-H (Log)
Y Parameter: FL2-H (Log)

Quad	Events	% Gated	% Total
UL	6714	86.10	67.14
UR	47	0.60	0.47
LL	491	6.30	4.91
LR	546	7.00	5.46

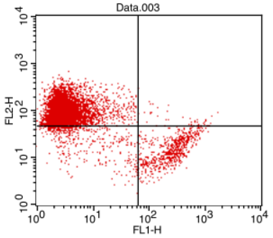

Quadrant Statistics

File: Data.003
Sample ID: LP1-DS-4h-20uMDHR
Gate: G1
Gated Events: 7626
Total Events: 10000
X Parameter: FL1-H (Log)
Y Parameter: FL2-H (Log)

Quad	Events	% Gated	% Total
UL	6415	84.12	64.15
UR	42	0.55	0.42
LL	543	7.12	5.43
LR	626	8.21	6.26

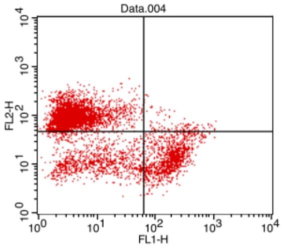

Quadrant Statistics

File: Data.004
Sample ID: LP1-DS-8h-20uMDHR
Gate: G1
Gated Events: 6404
Total Events: 10000
X Parameter: FL1-H (Log)
Y Parameter: FL2-H (Log)

Quad	Events	% Gated	% Total
UL	4055	63.32	40.55
UR	110	1.72	1.10
LL	1053	16.44	10.53
LR	1186	18.52	11.86

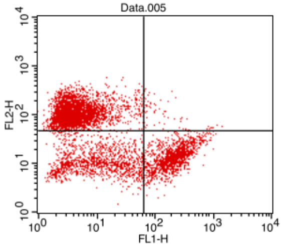

Quadrant Statistics

File: Data.005
Sample ID: LP1-DS-12h-20uMDHR
Gate: G1
Gated Events: 6262
Total Events: 10000
X Parameter: FL1-H (Log)
Y Parameter: FL2-H (Log)

Quad	Events	% Gated	% Total
UL	3649	58.27	36.49
UR	64	1.02	0.64
LL	1250	19.96	12.50
LR	1299	20.74	12.99

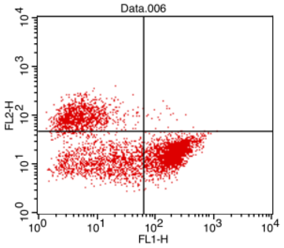

Quadrant Statistics

File: Data.006
Sample ID: LP1-DS-24h-20uMDHR
Gate: G1
Gated Events: 4395
Total Events: 10000
X Parameter: FL1-H (Log)
Y Parameter: FL2-H (Log)

Quad	Events	% Gated	% Total
UL	1118	25.44	11.18
UR	19	0.43	0.19
LL	1233	28.05	12.33
LR	2025	46.08	20.25

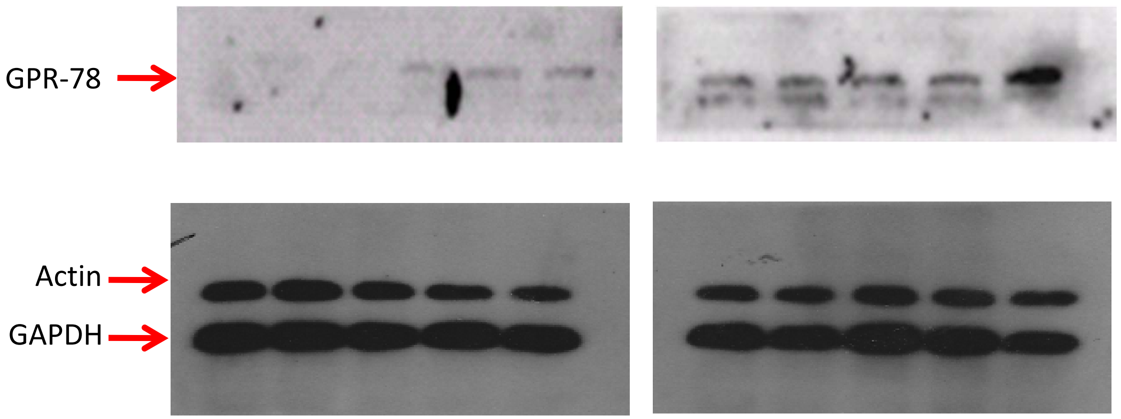

Fig. 5A We used the same blots for Bim (Fig. 3A) and the same Actin control was used.

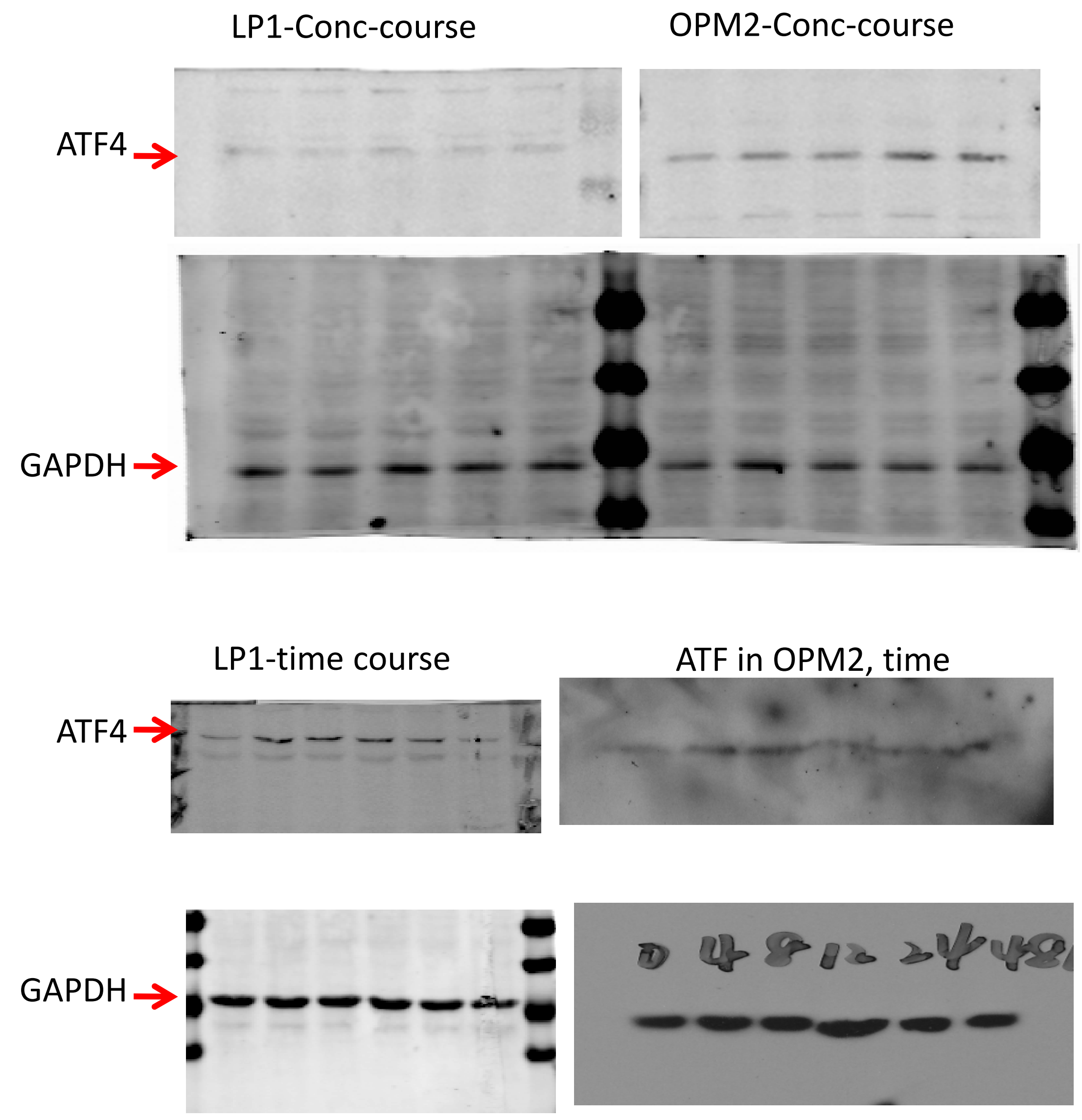
LP1-Conc-course
OPM2-Conc-course
ATF4
GAPDH
LP1-time course
ATF in OPM2, time
ATF4
GAPDH

Figure 5B

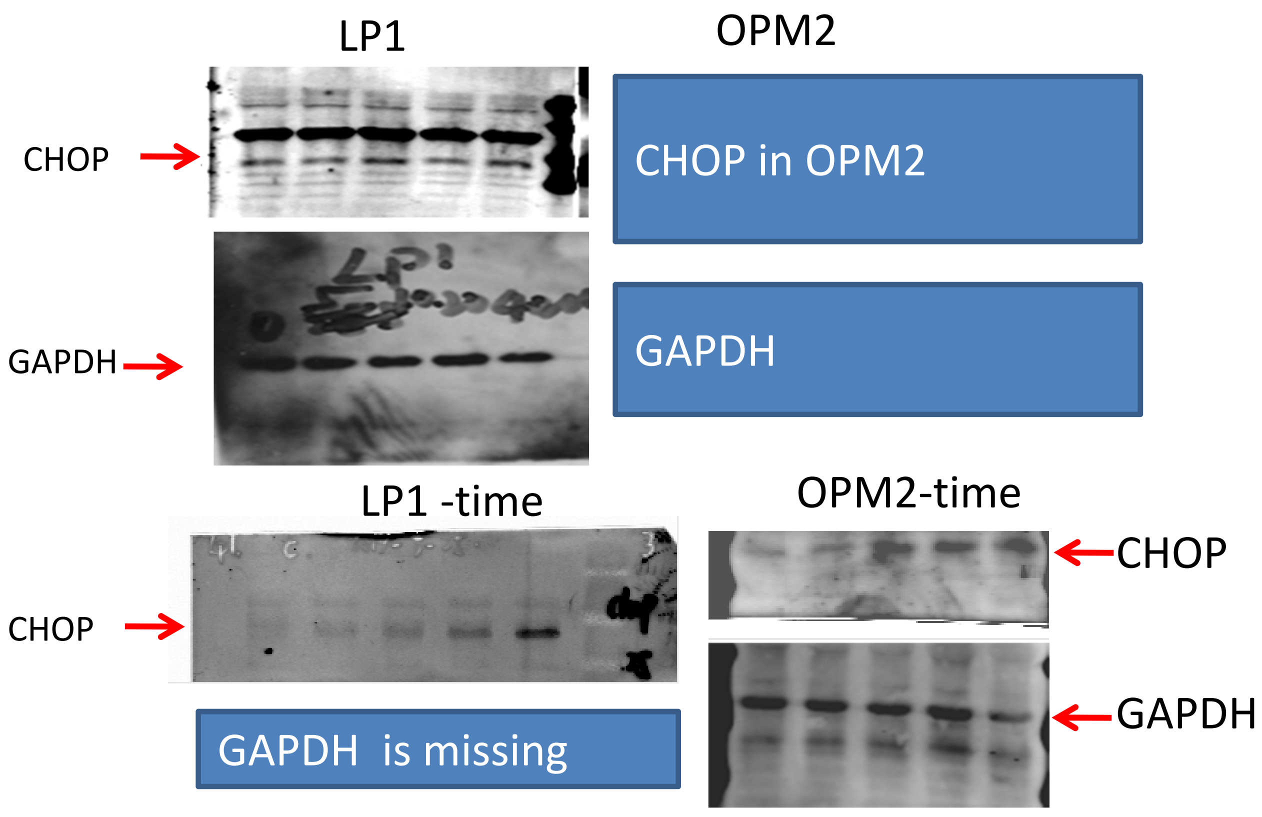
LP1
OPM2
CHOP
CHOP in OPM2
GAPDH
GAPDH
LP1 -time
OPM2-time
CHOP
CHOP
GAPDH is missing
GAPDH

Figure 5C

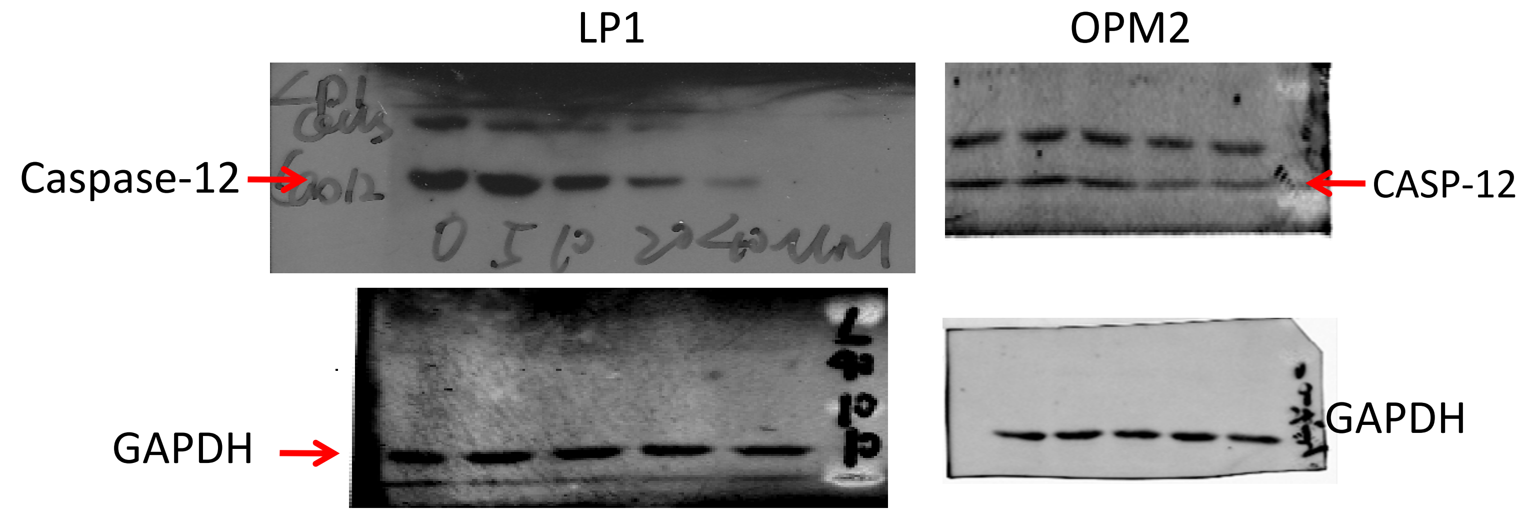

Figure 5D

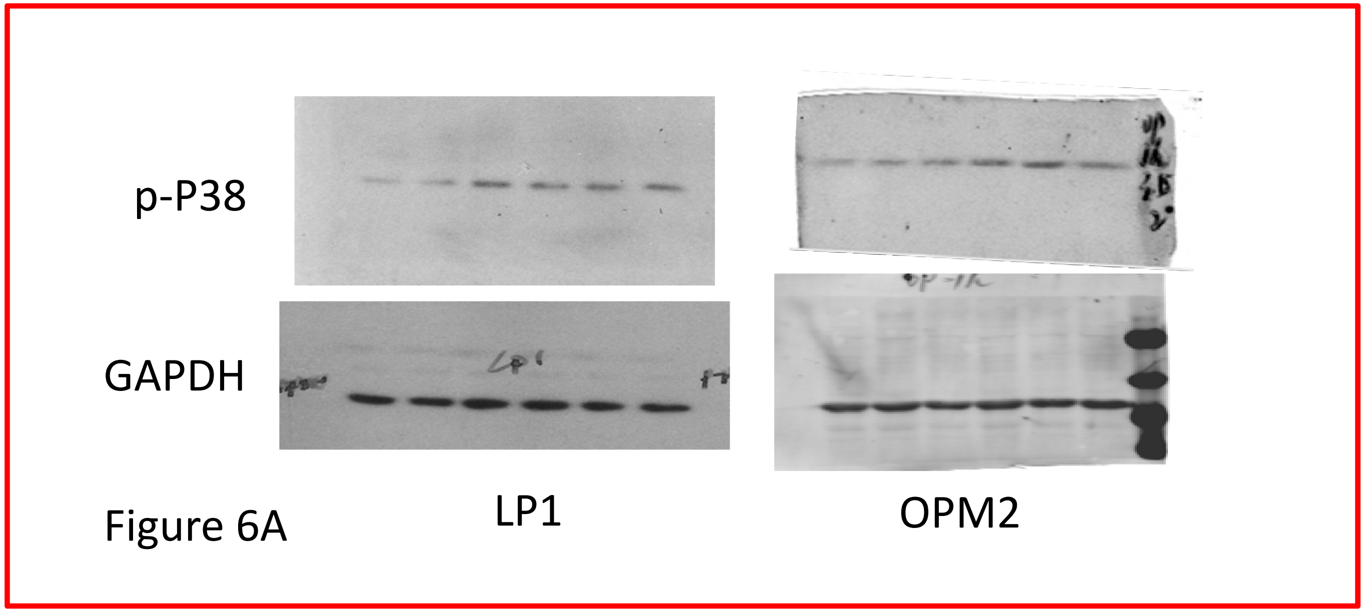

Figure 6A

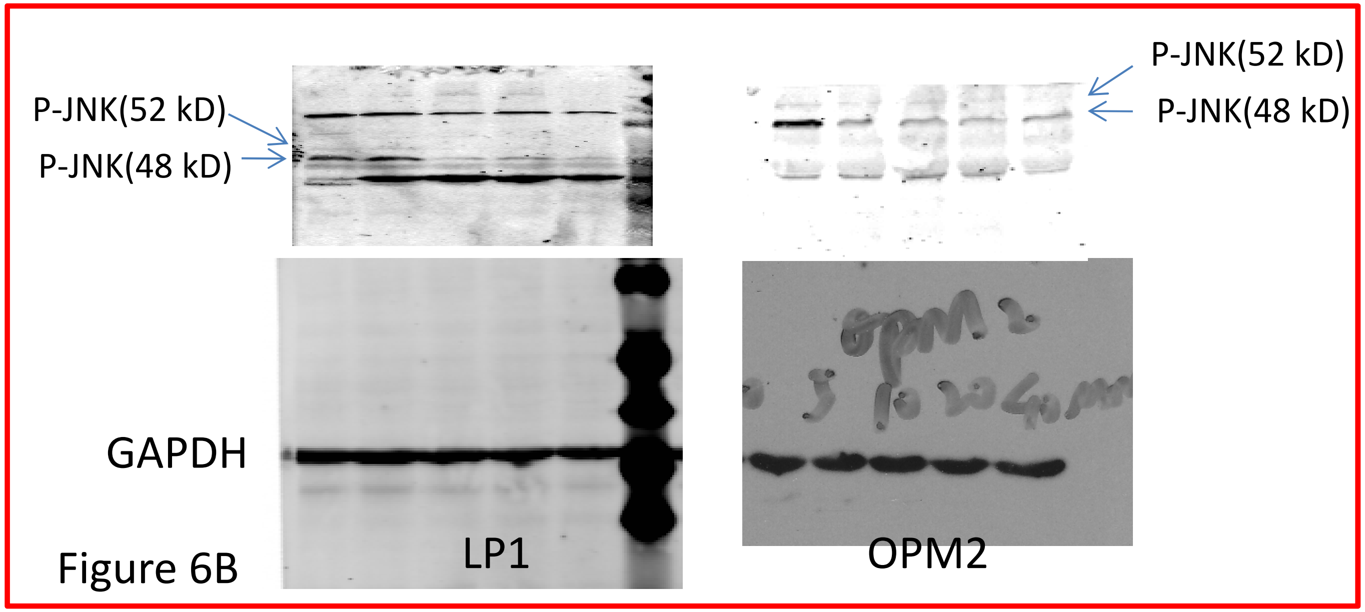

Figure 6B

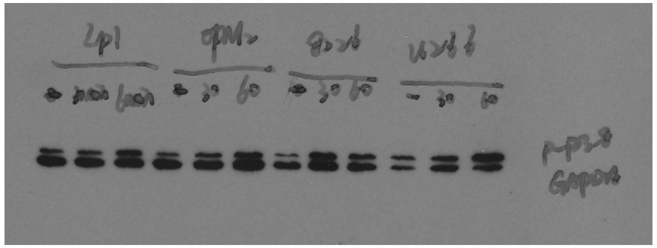

Figure 7A

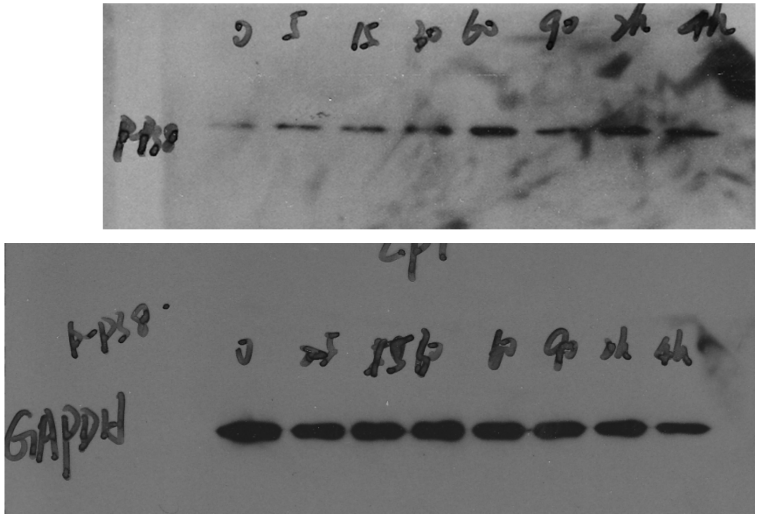

Figure 7B

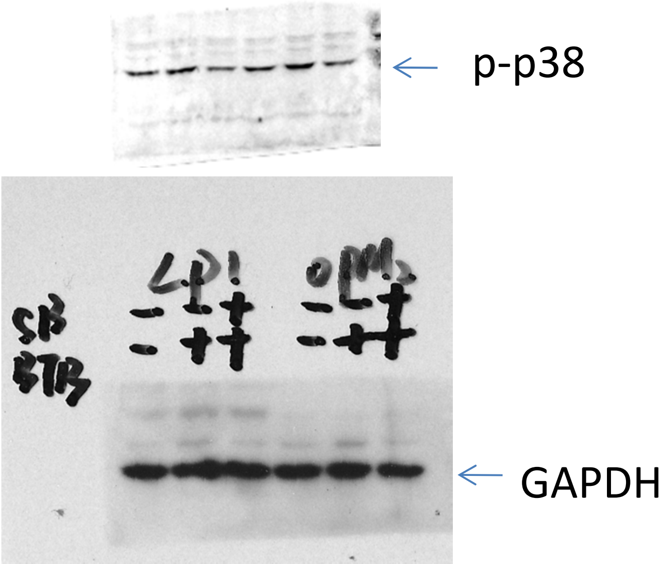

p-p38 was obtained from this blot with extended exposure.

Figure 7C

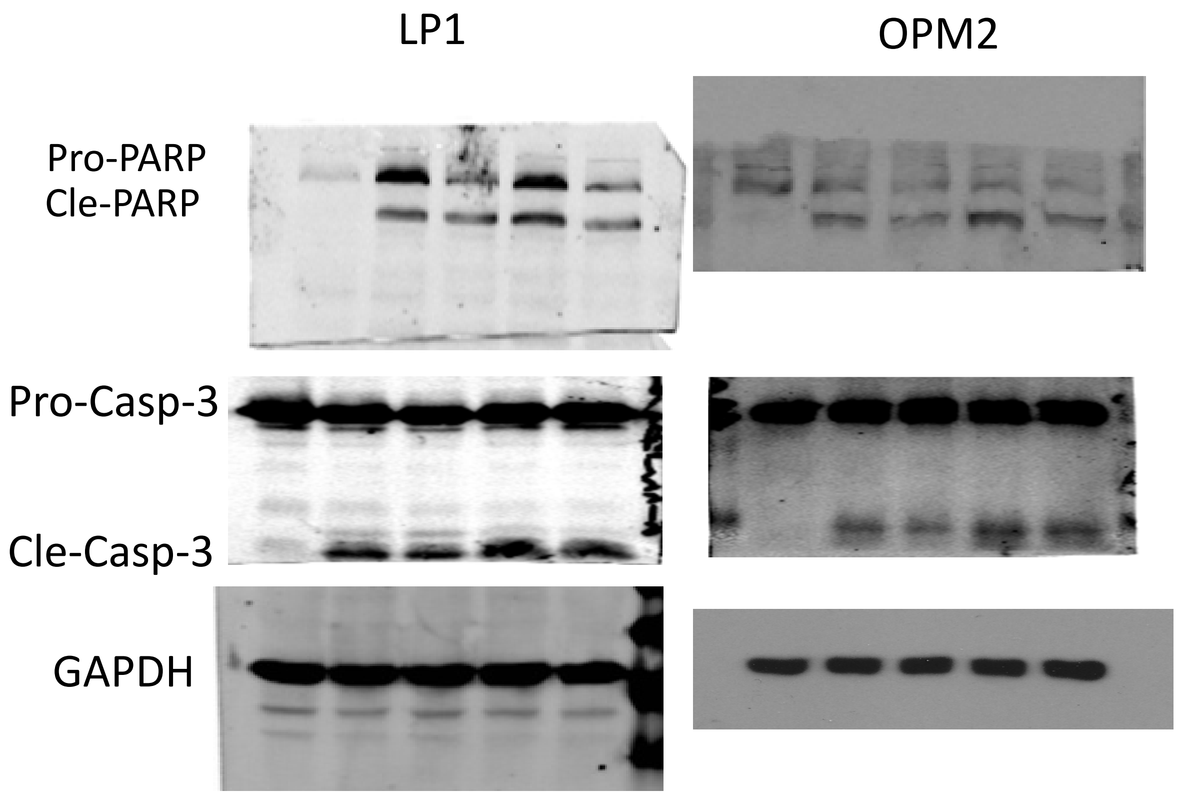

Figure 7D
