# Supplementary material for: Correction: The Natural Pesticide Dihydrorotenone Induces Human Plasma Cell Apoptosis by Triggering Endoplasmic Reticulum Stress and Activating p38 Signaling Pathway
Source: PLoS One. 2020 Jun 2;15(6):e0234162. doi: 10.1371/journal.pone.0234162 (PMC7266331; doi:10.1371/journal.pone.0234162)
Supplement: S2 File — (PDF) [file pone.0234162.s002.pdf]

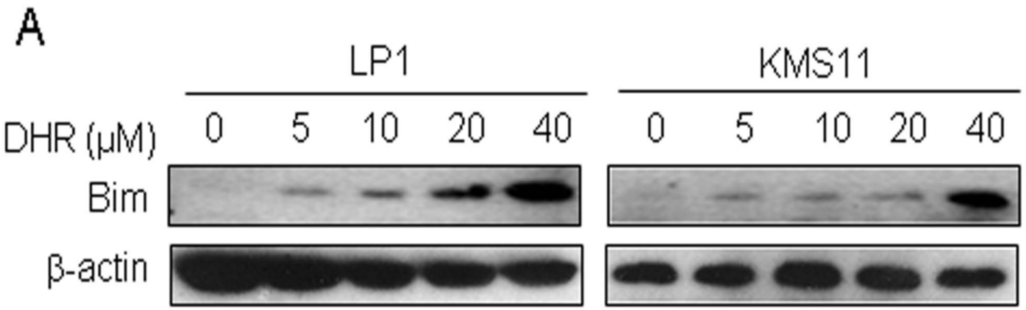

Bim →

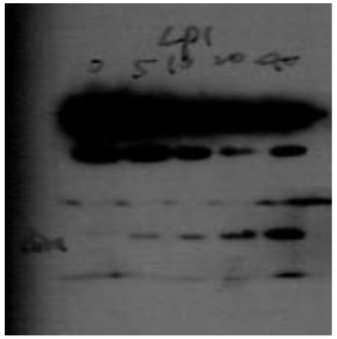

This film was not cut for individual WB

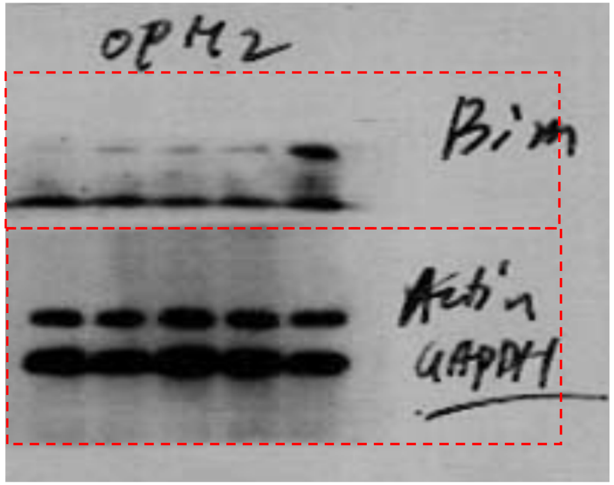

This film was cut for assays, one WB for Bim, another one for internal control of b-actin and GAPDH. Based on the original films, Bim was examined in LP1 and OPM2 (NOT KMS11) cells. There was an error in the figure composition.
