# Supplementary material for: Correction: The Natural Pesticide Dihydrorotenone Induces Human Plasma Cell Apoptosis by Triggering Endoplasmic Reticulum Stress and Activating p38 Signaling Pathway
Source: PLoS One. 2020 Jun 2;15(6):e0234162. doi: 10.1371/journal.pone.0234162 (PMC7266331; doi:10.1371/journal.pone.0234162)
Supplement: S3 File — (PDF) [file pone.0234162.s003.pdf]

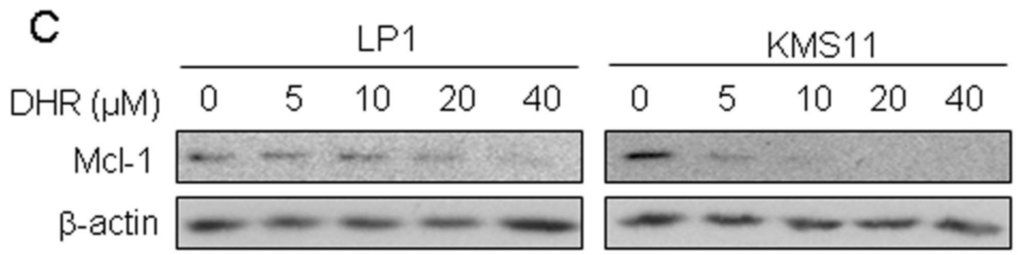

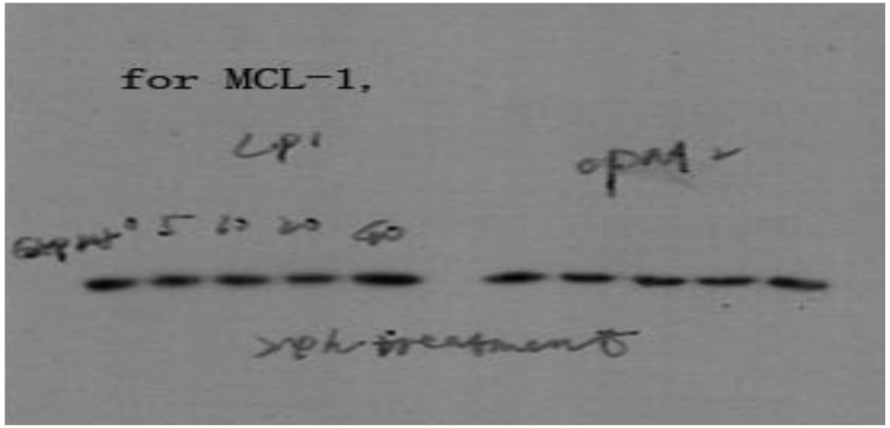

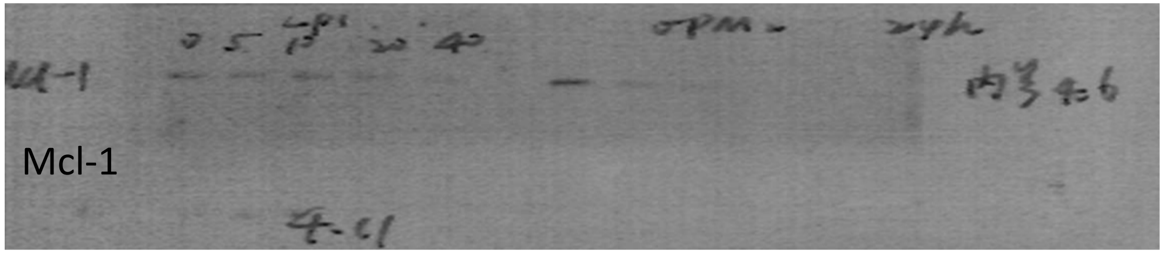

By check the original films, MCL-1 was evaluated in LP1 and OPM2 (NOT KMS11) cells. There was a mistake in the final figure composition. Therefore, we should make a correction for Figure 3. But the results will not affect the conclusion of the paper.
